# Heterologous Expression and Characterization of a Novel Polygalacturonase (DdpgA) from *Dickeya dadantii* DCE-01 and Its Application in Bioscouring of Ramie Fabric

**DOI:** 10.3390/polym18212618

**Published:** 2026-10-22

**Authors:** Ke Shi, Qi Yang, Guoguo Xi, Chen Chen, Ke Zheng, Yiyi Guo, Xiang Zhou, Qi Li, Tuo Lv, Shengwen Duan, Lifeng Cheng

**Affiliations:** 1 Institute of Bast Fiber Crops, Chinese Academy of Agricultural Sciences, Changsha 410205, China; 821012450485@caas.cn (K.S.); yangqi@caas.cn (Q.Y.); xiguoguo@caas.cn (G.X.); chenchen@caas.cn (C.C.); zhengke@caas.cn (K.Z.); 18630040948@163.com (Y.G.); 821012430018@caas.cn (X.Z.); lvtuo@caas.cn (T.L.); 2 Hunan Lerkam Biology Co., Ltd., Yueyang 414100, China; 127liqi@163.com

**Keywords:** *Dickeya dadantii*, polygalacturonase, enzymatic properties, ramie fabric, bioscouring

## Abstract

Efficient and eco-friendly bioscouring of ramie fabric is a critical step in the textile industry. To obtain polygalacturonase (DdpgA) suitable for the bioscouring of ramie fabric, a novel polygalacturonase gene (*ddpg*A) from the ramie-biodegumming strain *Dickeya dadantii* DCE-01 was subjected to bioinformatic analysis and heterologous expression. The recombinant plasmid pET28a-*ddpg*A was introduced into *E. coli* BL21(DE3), and the engineering strain BL21/pET28a-*ddpg*A was constructed; then induction conditions were optimized by response surface methodology. The recombinant DdpgA was purified and enzymatically characterized and further applied to the treatment of greige ramie fabric in combination with mannanase and xylanase. The results showed that the optimal pH of DdpgA was 5.0 and the optimal temperature was 55 °C; the enzyme was stable over the pH range of 3.0–6.0 and retained more than 50% of its activity after incubation at 55 °C for 60 min. DdpgA exhibited the highest catalytic activity toward sodium polygalacturonate. Mn^2+^ significantly activated DdpgA, whereas Ba^2+^, Pb^2+^, and Cu^2+^ inhibited its activity. Compound-enzyme scouring of ramie fabric formulated with DdpgA achieved a weight loss rate of 15.22%, a pectin removal rate of 51.28%, and a water-drop wetting time of less than 2 s; scanning electron microscopy confirmed the effective removal of gummy composites from the ramie fiber surface. This enzymatic scouring for ramie fabric avoids the strong alkali and high temperature required by conventional alkalic scouring, and thus represents an environmentally friendly alternative for ramie-fiber processing. Therefore, this study achieved high-level soluble expression of *ddpg*A in *E. coli* and obtained an acidic mesophilic polygalacturonase with demonstrated ramie bioscouring performance.

## 1. Introduction

Pectinases are a group of enzymes that degrade pectic substances. According to their modes of action on substrates, they are classified into four major types: protopectinase, polygalacturonase, pectin lyase, and pectin esterase [1]. Polygalacturonase (PG, EC 3.2.1.15) belongs to glycoside hydrolase family 28 (GH28) and specifically catalyzes the hydrolysis of α-1,4-galactosidic bonds in the homogalacturonan backbone, generating galacturonic acid monomers or oligomers, and it is the most common type of pectinase [2,3].

Polygalacturonases are widely distributed in plants, fungi, and bacteria and play important roles in fruit softening, plant pathogenesis, and plant cell wall degradation [4,5,6]. At the industrial level, PGs have been extensively applied in juice clarification and extraction, winemaking, degumming and scouring of textile fibers, treatment of papermaking wastewater, and removal of the mucilage from coffee and cocoa beans, conferring considerable economic value [7,8,9]. In recent years, with the promotion of biologic manufacturing, the enzymatic approach has attracted increasing attention in the textile industry owing to its mild reaction conditions, environmental friendliness, and low energy consumption. Conventional chemical scouring of bast fibers relies on strong acids, strong alkalis, and high-temperature treatments, which suffer from high energy consumption, severe pollution, and serious fiber damage [10,11]. Polygalacturonase-based bioscouring, synergistic hemi-cellulase (mannanase and xylanase), removes non-cellulosic substances from the fiber surface under mild conditions, effectively preserving the mechanical properties of the fibers while reducing the consumption of chemicals and the discharge of wastewater [12,13].

However, commercially available polygalacturonases still suffer from low activity, insufficient thermostability, and a narrow applicable pH range; in particular, highly efficient PG preparations dedicated to bast-fiber processing are lacking. Moreover, most existing PGs are of fungal origin and are produced mainly in *Pichia pastoris* expression systems, which involve long fermentation cycles and high costs [14]. In contrast, the *E. coli* expression system offers a well-characterized genetic background, rapid growth, low fermentation cost, and high expression levels, making it an ideal host for the high-level expression of prokaryotic enzymes [15]. Therefore, the discovery of novel high-activity polygalacturonase genes and the achievement of their high-level soluble expression in *E. coli* are of great ecological and economic benefits [16,17].

Our group previously isolated the highly efficient bast-fiber-degumming strain *Dickeya dadantii* DCE-01 from the natural environment, which can complete ramie degumming within 6 h [18]. Whole-genome sequencing and functional annotation revealed that this strain harbors 21 enzyme-coding genes related to bast-fiber degumming and scouring, including the polygalacturonase gene, the xylanase gene, and the mannanase gene and so on [19,20]. In this study, *ddpg*A will be heterologously expressed, and the soluble expression of DdpgA will be improved by optimizing the induction conditions to achieve high production. Furthermore, its enzymatic properties will be systematically characterized, and its performance in the bioscouring of greige ramie fabric will be evaluated.

## 2. Materials and Methods

### 2.1. Bacterial Strains and Expression Vector

*E. coli* DH5α and *E. coli* BL21(DE3) competent cells were purchased from Sangon Biotech (Shanghai, China). The target gene *ddpg*A (GenBank: PQ219936), annotated as a polygalacturonase gene in the genome of *D. dadantii* DCE-01, was codon-optimized and synthesized by Sangon Biotech; the recombinant plasmid pET-28a-*ddpg*A was constructed using the kanamycin (Kan)-resistant vector pET-28a for the heterologous expression of polygalacturonase.

### 2.2. Media and Reagents

LB liquid medium: yeast extract (5 g/L), tryptone (10 g/L), and NaCl (10 g/L), adjusted to pH 7.5 with 5 mol/L NaOH and sterilized at 121 °C for 20 min.

LB solid medium: LB liquid medium supplemented with 1.5% (*w*/*v*) agar, sterilized at 121 °C for 20 min.

Sodium polygalacturonate (purity ≥ 75%), apple pectin, orange pectin, pectin (DE ≥ 85%), pectin (DE 55–70%), and D-galacturonic acid were purchased from Sigma-Aldrich (Darmstadt, Germany); mannanase, xylanase, and polygalacturonase were obtained from Hunan Lerkam Biology Co., Ltd. (Yueyang, China); a plasmid extraction kit was purchased from Tiangen Biotech (Beijing, China); ultrafiltration tubes were purchased from Millipore(Darmstadt, Germany). DNS reagent and analytical-grade reagents were purchased from Shanghai Macklin Biochemical Co., Ltd. (Shanghai, China).

### 2.3. Bioinformatics Analysis

The theoretical molecular weight, isoelectric point, instability index, grand average of hydropathicity, and glycosylation sites of DdpgA were predicted using ExPASy ProtParam (https://web.expasy.org/protparam/, accessed on 10 September 2025) and DTU Health Tech (https://services.healthtech.dtu.dk/, accessed on 10 September 2025). The amino acid sequence of DdpgA was used as a query for BLASTp searching against the NCBI database (http://www.ncbi.nlm.nih.gov/, accessed on 12 September 2025), and similar sequences were downloaded. Multiple sequence alignment was performed using ClustalW (https://www.genome.jp/tools-bin/clustalw, accessed on 25 August 2025) and ESPript 3.2 (http://espript.ibcp.fr/ESPript/cgi-bin/ESPript.cgi, accessed on 25 August 2025). The phylogenetic tree was constructed using MEGA 12.0. The three-dimensional structure was analyzed by homology modeling using SWISS-MODEL (https://swissmodel.expasy.org/, accessed on 17 June 2026) and PyMOL3.1.

### 2.4. Construction of the Engineering Strain BL21/pET-28a-ddpgA and Induction of DdpgA

The *ddpg*A gene sequence was codon-optimized according to the codon preference of *E. coli* without altering the original amino acid sequence. The optimized gene was cloned into the multiple cloning site of the pET-28a expression vector with a 6× His tag fused at the C-terminus, generating the recombinant plasmid pET-28a-*ddpg*A.

1 μL of the recombinant plasmid was added to 100 μL of BL21(DE3) competent cells, incubated on ice for 30 min, heat-shocked at 42 °C for 90 s, and immediately chilled on ice for 2 min. Then, 500 μL of LB liquid medium was added, and the cells were recovered at 37 °C and 200 r/min for 45–60 min. A 100 μL aliquot of the cell suspension was spread on LB agar plates containing 50 μg/mL Kan and incubated at 37 °C for 16–20 h. A positive single colony was inoculated into 5 mL of LB liquid medium containing 50 μg/mL Kan and cultivated overnight at 37 °C and 220 r/min. The culture was transferred at 1% (*v*/*v*) into 100 mL of fresh LB medium and grown at 37 °C and 220 r/min until the *OD*_600_ reached 0.4–0.6. IPTG (isopropyl β-D-1-thiogalactopyranoside) was added to a final concentration of 0.5 mmol/L, and the culture was further induced at 16 °C for 16 h. The cells were harvested by centrifugation at 8000 r/min for 10 min at 4 °C, and the expression was examined by SDS-PAGE.

### 2.5. Assay of Polygalacturonase Activity

PG activity was determined by the 3,5-dinitrosalicylic acid (DNS) colorimetric method [21], using 0.33% (*w*/*v*) sodium polygalacturonate dissolved in Na_2_HPO_4_–citrate buffer (pH 5.0) as the substrate. An appropriately diluted enzyme solution was added to the substrate solution preheated to 55 °C and reacted exactly at 55 °C for 3 min. Then, 2.5 mL of DNS reagent was added; the mixture was heated in a boiling water bath for 5 min, cooled immediately in an ice-water bath, and brought to 15 mL with double-distilled water. The absorbance was measured at 540 nm, with heat-inactivated enzyme solution as the blank. One unit (U) of enzyme activity was defined as the amount of enzyme that catalyzed the formation of 1 μmol of D-galacturonic acid per minute under the above conditions.

### 2.6. Optimization of the Induction Conditions of BL21/pET-28a-ddpgA

#### 2.6.1. Single-Factor Experiments

Induction temperature: the engineered strain was cultivated to the logarithmic phase, induced with IPTG at a final concentration of 0.5 mmol/L, and cultured at a temperature gradient of 16–40 °C for 8 h, followed by the determination of DdpgA activity.

IPTG concentration: the engineered strain was cultivated to the logarithmic phase and induced with IPTG at final concentrations ranging from 0.1 to 1.4 mmol/L at 37 °C for 8 h, followed by the determination of DdpgA activity.

Induction time: the engineered strain was cultivated to the logarithmic phase, induced with IPTG at a final concentration of 0.5 mmol/L at 25 °C for 4–32 h, and the DdpgA activity was determined.

#### 2.6.2. Response Surface Optimization

On the basis of the single-factor experiments, a three-factor, three-level Box–Behnken design (BBD) was employed, with induction temperature (A: 20, 25, and 30 °C), IPTG concentration (B: 0.3, 0.5, and 0.7 mmol/L), and induction time (C: 16, 20, and 24 h) as the independent variables. Seventeen experimental runs (including five center points) were designed, with enzyme activity (U/mL) as the response value. Regression analysis and model fitting were performed using Design-Expert 13.0 software to predict the optimal induction conditions, which were then verified experimentally. The measured enzyme activity was compared with that before optimization.

### 2.7. Purification of the Recombinant DdpgA

The harvested cells were washed once with PBS buffer (pH 7.5) and resuspended in 10 mL of the same buffer. The cells were disrupted by sonication (3 s on and 5 s off at 200 W for 30 min in total) and centrifuged at 8000 r/min for 30 min, and the supernatant was transferred to a new 15 mL centrifuge tube. The supernatant was purified by Ni-NTA affinity chromatography [22] and concentrated using ultrafiltration tubes with a 30 kDa molecular-weight cut-off.

Purified DdpgA was analyzed by SDS-PAGE, and the protein concentration was determined by the Bradford method.

### 2.8. Enzymatic Characterization of DdpgA

#### 2.8.1. Optimal pH and pH Stability

The activity of the purified DdpgA was assayed at 55 °C in Na_2_HPO_4_–citrate buffers (pH 3.0–8.0) containing 0.33% (*w*/*v*) sodium polygalacturonate, and the relative activity was calculated with the highest activity set as 100%. For pH stability, the purified DdpgA was incubated in buffers of pH 3.0–8.0 at 37 °C for 1 h, and the residual enzymatic activity was determined under the optimal conditions, with the activity of the untreated enzyme set as 100%.

#### 2.8.2. Optimal Temperature and Thermostability

Optimal temperature: the enzyme activity was assayed at temperatures ranging from 30 to 80 °C under the optimal pH, and the relative activity was calculated with the highest activity set as 100%. For thermostability, the purified enzyme was incubated at 40, 45, 50, 55, 60, or 65 °C and sampled periodically within 0–120 min; each sample was cooled immediately in an ice-water bath, and the residual activity was determined under the optimal conditions, with the activity of the untreated enzyme set as 100%.

#### 2.8.3. Effects of Metal Ions and Chemical Reagents

Metal ions (Mn^2+^, Zn^2+^, Ni^2+^, Mg^2+^, Cr^3+^, Ca^2+^, Fe^3+^, Ba^2+^, Pb^2+^, and Cu^2+^) and chemical reagents (ethylenediaminetetraacetic acid, sodium dodecyl sulfate, and β-mercaptoethanol) were added separately to the reaction system at a final concentration of 1.0 mmol/L. The residual enzyme activity was determined under the optimal reaction conditions, and the relative activity was calculated with the activity of the control without any additive set as 100%.

#### 2.8.4. Substrate Specificity

Sodium polygalacturonate, pectins with different degrees of esterification (DE 29%, 49%, 55–70%, and ≥85%), apple pectin, orange pectin, and CMC-Na(carboxymethyl cellulose sodium) were used as substrates, and the enzyme activity was determined under the optimal reaction conditions. The activity toward sodium polygalacturonate was set as 100%.

#### 2.8.5. Kinetic Parameters

Sodium polygalacturonate solutions of different concentrations (1–10 mg/mL) were prepared, and the initial reaction rates at the respective substrate concentrations were determined under the optimal reaction conditions. The Michaelis–Menten equation was fitted by nonlinear regression using Origin2024 software to calculate Km, Vmax, kcat, and kcat/Km.

#### 2.8.6. MALDI-TOF MS Analysis of the Hydrolysis Products

The purified DdpgA was incubated with 10 mL of 0.5% (*w*/*v*) polygalacturonic acid at 40 °C and pH 5.0 for 12–14 h, followed by rapid inactivation by boiling. The pretreated hydrolysate supernatant was loaded into a 3 kDa ultrafiltration tube and centrifuged at 4000× *g* at 4 °C until 400–500 μL of concentrate remained in the inner tube. The filtrate was collected, freeze-dried, and analyzed by MALDI-TOF MS (Bruker autoflex™ speed, Billerica, MA, USA).

### 2.9. Bioscouring of Ramie Fabric

#### 2.9.1. Enzymatic Scouring and Conventional Alkaline Scouring

Enzymatic scouring: the treatment bath was prepared at a bath ratio of 1:15 (fabric-to-liquor) and 50 °C, an appropriate amount of the purified enzyme was added, and the pH was adjusted to the set value. The ramie fabric, previously dried to constant weight, was immersed in the treatment bath and shaken at the set temperature for the set time. After treatment, the fabric was removed, rinsed with hot water at 95 °C for 2 min (two cycles) and then with cold water for 2 min (two cycles), and finally dried to constant weight at 105 °C and weighed.

Alkaline scouring: NaOH 8 g/L, pH 9–10, bath ratio 1:30, at 95 °C for 90 min. The subsequent washing and drying steps were the same as those for enzymatic scouring.

#### 2.9.2. Evaluation of Scouring Efficiency

Water-drop wetting time: the fabric was fixed on a flat surface with both sides freely suspended, a small drop of deionized water was placed on the fabric surface, and the time required for the drop to penetrate the fabric completely was recorded. Five random positions were measured and averaged.

Weight loss rate: The fabric was dried to constant weight at 105 °C before and after treatment and weighed. The weight loss rate was calculated as follows:

$$\mathrm{WL}\left(\%\right)=\frac{m_{1}-m_{2}}{m_{1}}\times \;100\%$$

where *m*_1_ is the mass of the fabric before treatment and *m*_2_ is the mass of the fabric after treatment.

Pectin removal rate: The pectin content of the fabric was determined by the carbazole–sulfuric acid method. A 1.5 g portion of the treated ramie fabric was cut into pieces of approximately 1 cm^2^ and extracted with 100 mL of 0.5% (*w*/*v*) ammonium oxalate solution in a shaking water bath at 98 °C for 2 h, followed by hot filtration. One milliliter of the filtrate was mixed with 6 mL of concentrated sulfuric acid; after mixing and cooling, the mixture was heated in a boiling water bath for 10 min and cooled to room temperature, and 0.5 mL of 0.15% (*w*/*v*) carbazole solution was added. After standing at room temperature in the dark for 30 min, the absorbance was measured at 530 nm. The pectin content was calculated from the D-galacturonic acid standard curve as follows:

$$w=\frac{\mathrm{cvk}}{m\times 10^{6}}$$

$$G\left(\%\right)=\frac{w_{1}-w_{2}}{w_{1}}\times \;100\%$$

where *w* is the pectin content, *c* is the concentration, *v* is the volume of the ammonium oxalate solution, *k* is the dilution factor, and *m* is the weight; *w*_2_ is the pectin content of the treated ramie fabric, *w*_1_ is that of the original ramie fabric, and *G* is the pectin removal rate.

Surface morphology of the fibers: The dried fabric samples were cut to an appropriate size, mounted on specimen stubs with conductive adhesive, and sputter-coated with gold. The surface morphology of the fibers was observed using an SEM (SU3500, HITACHI, Tokyo, Japan) at an accelerating voltage of 3 kV.

## 3. Results

### 3.1. Bioinformatics Analysis of DdpgA

#### 3.1.1. Sequence Analysis

The DdpgA from *D. dadantii* DCE-01 belongs to glycoside hydrolase family GH28. The physicochemical properties of DdpgA were predicted and shown in Table 1. DdpgA consists of 602 amino acid residues, with a predicted molecular weight of 64.25 kDa and a theoretical pI of 7.14. An N-terminal signal peptide of 27 amino acids was identified, indicating that DdpgA is a secreted protein. In addition, six N-glycosylation sites, eleven O-glycosylation sites, and a pair of cysteines were predicted, suggesting that DdpgA is rich in post-translational glycosylation modification sites.

**Figure 1:**
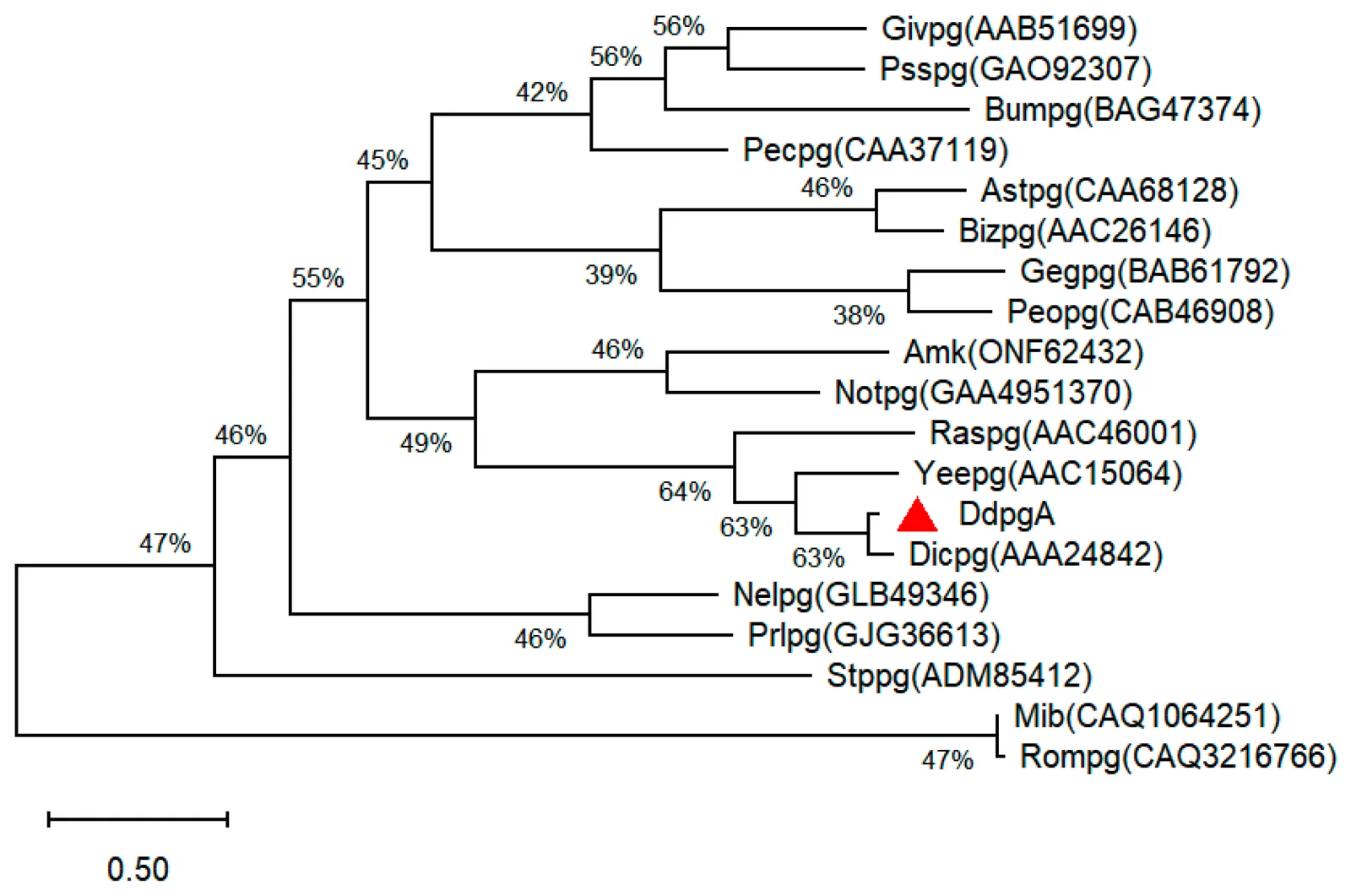
Phylogenetic analysis of PGs from bacteria. The red triangle highlights DdpgA from *D. dadantii* DCE-01 characterized in this study.

**Figure 2:**
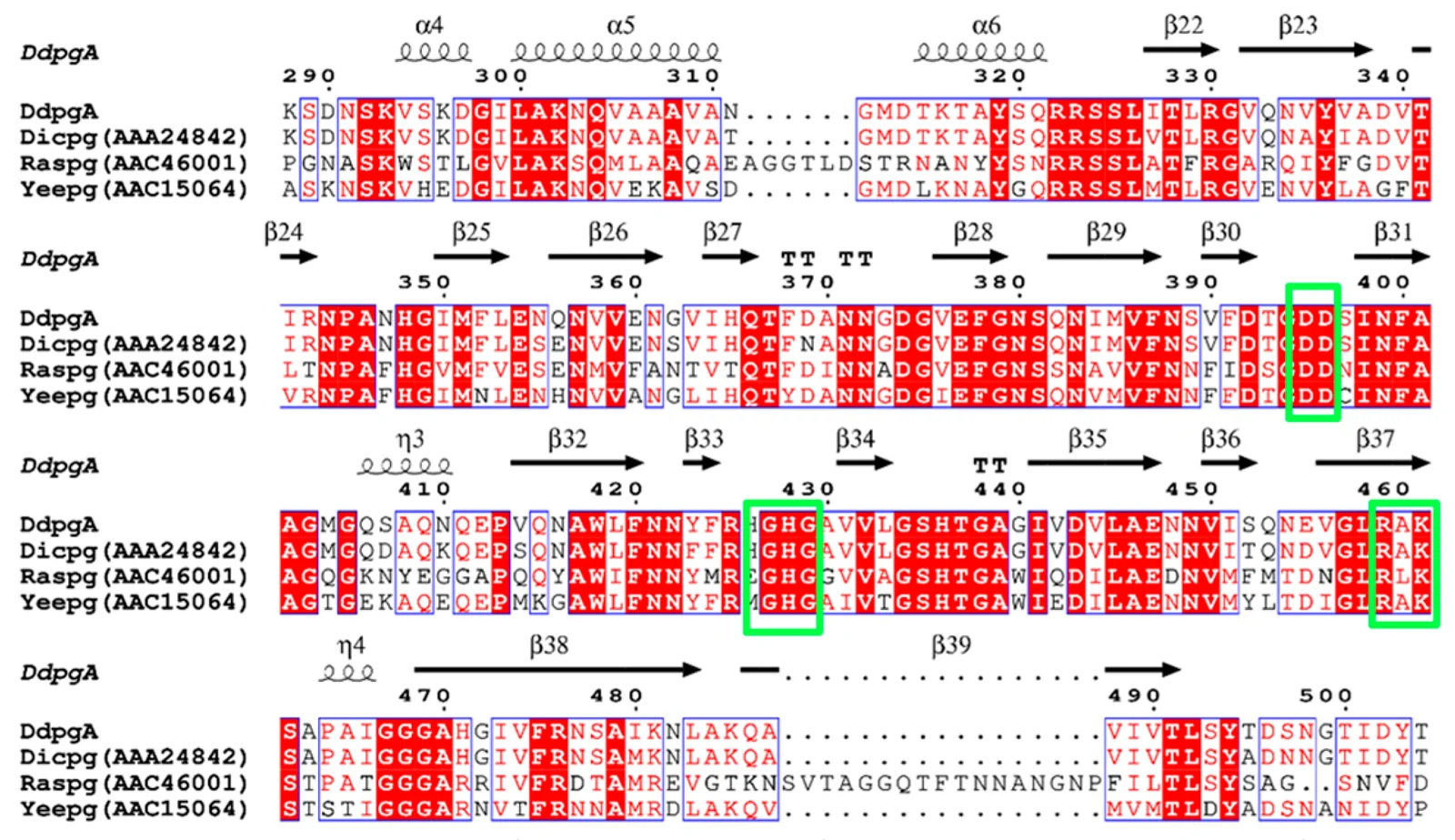
Multiple sequence alignment and domain analysis of DdpgA. (The conserved polygalacturonase domain is marked by green boxes; red background indicates identical residues; blue boxes indicate conserved similarity.).

Polygalacturonases are classified into exo- and endo-types. Multiple sequence alignment (ClustalW) was performed, and a phylogenetic tree was constructed by the maximum likelihood method using MEGA 12.0 to compare the DdpgA of *D. dadantii* DCE-01 with PGs from other bacteria (Figure 1). The results showed that DdpgA is closely related to the exo-polygalacturonases DicpgA (GenBank: AAA24842) from *Dickeya chrysanthemi*, Yeepg (GenBank: AAC15064) from *Yersinia enterocolitica*, and Raspg (GenBank: AAC46001) from *Ralstonia solanacearum* K60. Aligning the four amino acid sequences (Figure 2), results showed that DdpgA contains the highly conserved sequence motifs of PGs, namely 95DD, 427GHG, and 459RAK.

#### 3.1.2. Structural Modeling and Analysis

**Figure 3:**
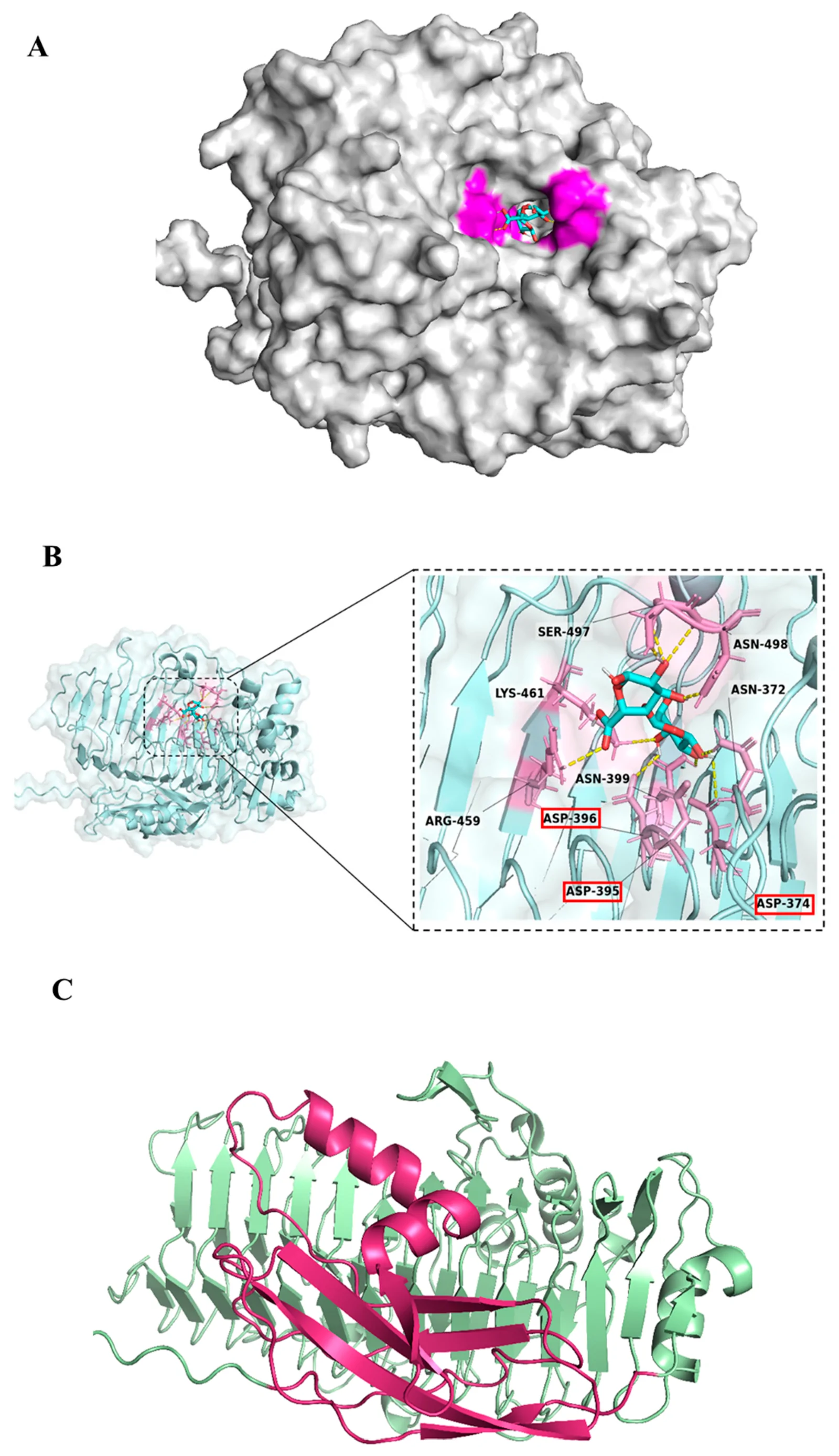
Three-dimensional structure, ligand docking, and domain architecture of DdpgA. (**A**) Surface model showing the ligand embedded in the catalytic pocket (pink shading indicates the catalytic pocket); (**B**) The catalytic triad Asp374-Asp395-Asp396 and other key amino acid residues, with yellow dashed lines indicating hydrogen bonds, pink highlighting key residues, and the galacturonic acid dimer ligand shown in stick representation (cyan for carbon atoms, red for oxygen atoms); (**C**) The N-terminal FN3 domain shown in red.

In DdpgA, the galacturonic acid dimer ligand was stably embedded in the catalytic pocket (Figure 3A), with a binding energy of −8.8 kcal/mol and an RMSD < 2 Å, indicating a stable and convergent binding mode. All three catalytic residues were within 3.2 Å of the ligand, indicating that the ligand was precisely positioned at the catalytic center, with the glycosidic bond aligned exactly against the catalytic triad Asp374-Asp395-Asp396 (Figure 3B). Arg459 and Lys461, which belong to the RAK motif, were located 2.65 Å and 2.7 Å, respectively, from the ligand and participated in the recognition of the substrate carboxyl group by forming a salt bridge with the C6-COOH of pectic acid, a key feature for the recognition of acidic substrates by GH28 enzymes. A dense hydrogen-bond network was formed between the ligand and the receptor, involving Asn372 (3.08 Å), Asp374 (2.87 Å), Asp396 (3.06 Å), Asn399 (2.66 Å), Ser497 (1.77 Å), and Asn498 (2.94 Å).

Domain annotation of the DdpgA amino acid sequence using InterProScan (https://www.ebi.ac.uk/interpro/about/interproscan/, accessed on 15 March 2026) identified an FN3 domain in the N-terminal region (residues 32–149) of DdpgA (Figure 3C).

### 3.2. Expression and Purification of the Recombinant DdpgA

**Figure 4:**
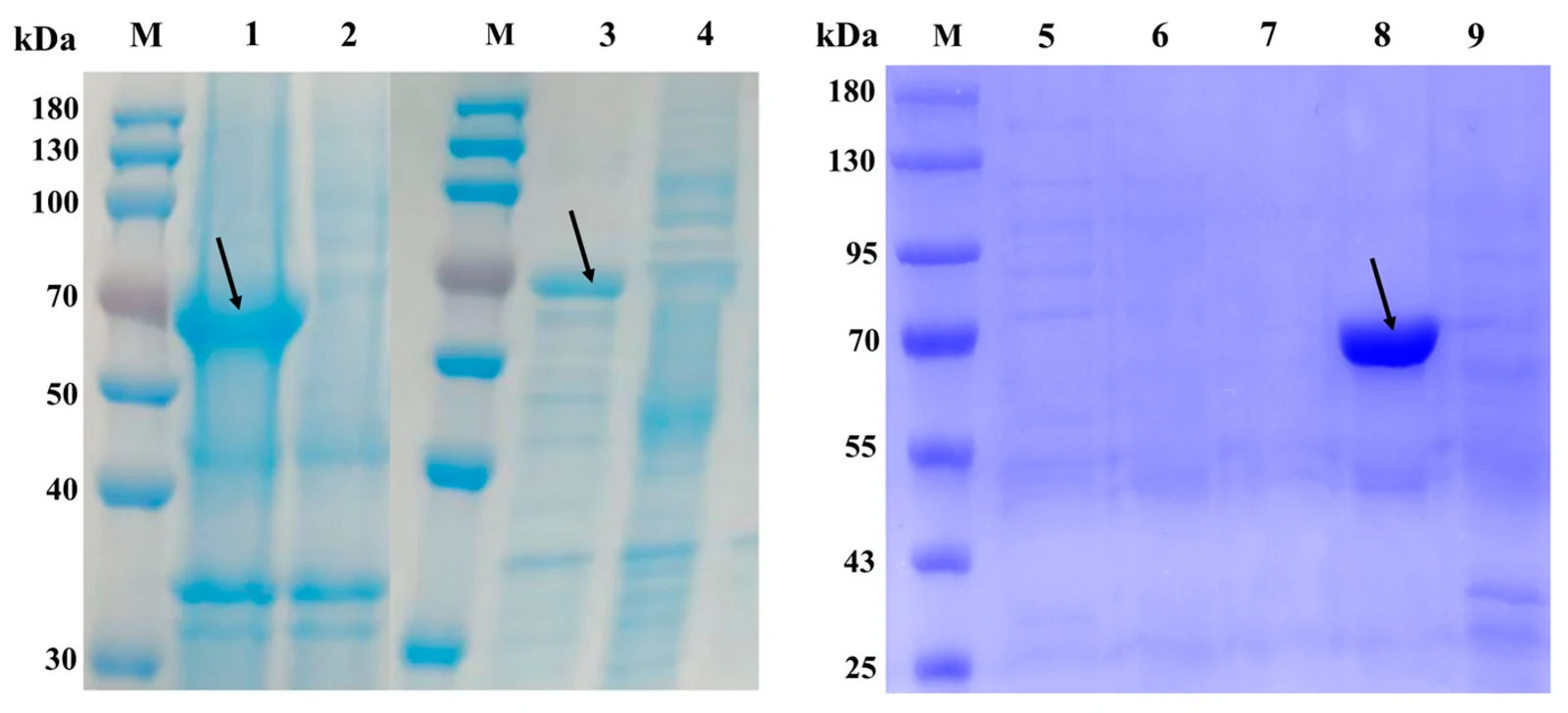
SDS-PAGE of DdpgA expression and purification. M: protein molecular weight marker; 1: whole-cell proteins of BL21/pET28a*-ddpg*A; 2: whole-cell proteins of BL21/pET28a (negative control); 3: soluble supernatant of BL21/pET28a-*ddpg*A; 4: soluble supernatant of BL21/pET28a (negative control); 5: elution fraction with 20 mM imidazole; 6: elution fraction with 40 mM imidazole; 7: elution fraction with 80 mM imidazole; 8: elution fraction with 400 mM imidazole; 9: flow-through fraction. Arrows indicate the target recombinant DdpgA protein band (64 kDa).

The engineered strain BL21/pET-28a-*ddpg*A was induced, and then DdpgA was purified by Ni-NTA affinity chromatography. As shown in Figure 4, a distinct band was observed at approximately 64.2 kDa for the purified protein, consistent with its theoretical molecular weight (64.25 kDa). The expression level of the recombinant protein was approximately 300 mg/L, and the specific activity reached 523.3 U/mg.

### 3.3. Optimization of the Induction Conditions for BL21/pET-28a-ddpgA

#### Response Surface Methodology

Preliminary single-factor experiments showed that the induction temperature, IPTG concentration, and induction time significantly affected the soluble expression of DdpgA in *E. coli*. Therefore, based on the results of the preliminary experiments, these three factors—induction temperature (20, 25, and 30 °C), IPTG concentration (0.3, 0.5, and 0.7 mmol/L), and induction time (16, 20, and 24 h)—were selected to construct a three-factor, three-level Box–Behnken response surface model, with enzyme activity as the response value. The experimental design and results are shown in Table 2. The data in Table 2 were fitted and analyzed using Design-Expert 13 software, yielding the following second-order polynomial regression equation in terms of coded values: Y = 321.58 + 11.83A − 17.48B + 4.64C + 13.87AB − 14.00AC − 3.89BC − 61.17A^2^ − 8.82B^2^ − 13.37C^2^. Analysis of variance (Table 3) showed that the model F-value was 48.02 with *p* < 0.0001, while the lack-of-fit *p* > 0.05 (not significant), indicating that the model was reliable. The R^2^ of 0.9841 and the adjusted R^2^ (R^2^Adj) of 0.9636 indicated a good model fit, explaining 96.36% of the variation in the response values. The coefficient of variation was 2.58%, indicating good reproducibility and feasibility of the experiments. Based on the F-values, the order of influence on enzyme production by induction was B > A > C. Among the model terms, the linear terms A and B, the interaction terms AB and AC, and the quadratic terms A^2^, B^2^, and C^2^ significantly affected enzyme production (*p* < 0.05).

**Figure 5:**
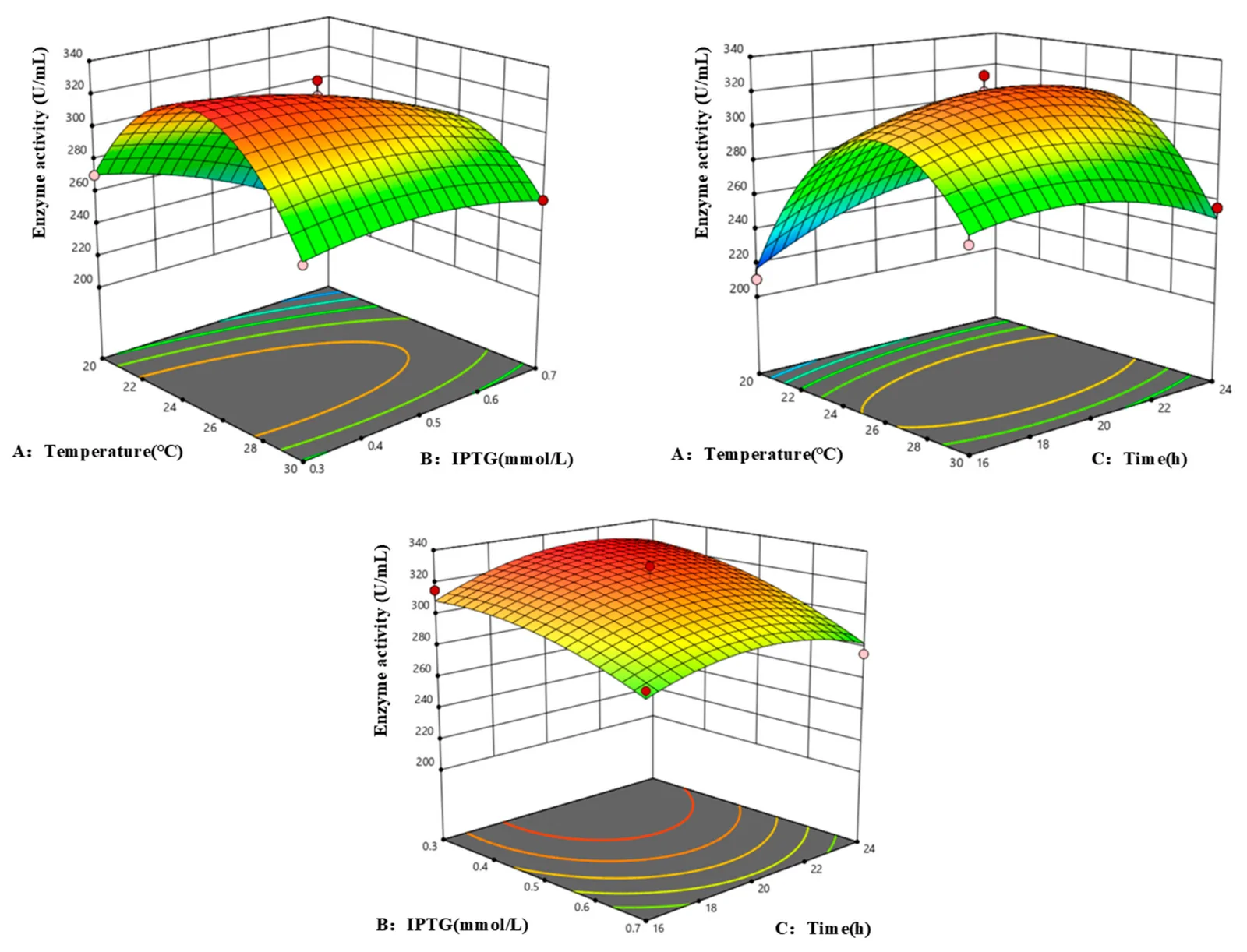
Response surface analysis of induction conditions on intracellular DdpgA activity. The color gradient (from blue to red) represents increasing enzyme activity; the contour lines on the base plane indicate equal activity levels; and the dots represent the actual experimental design points.

**Figure 6:**
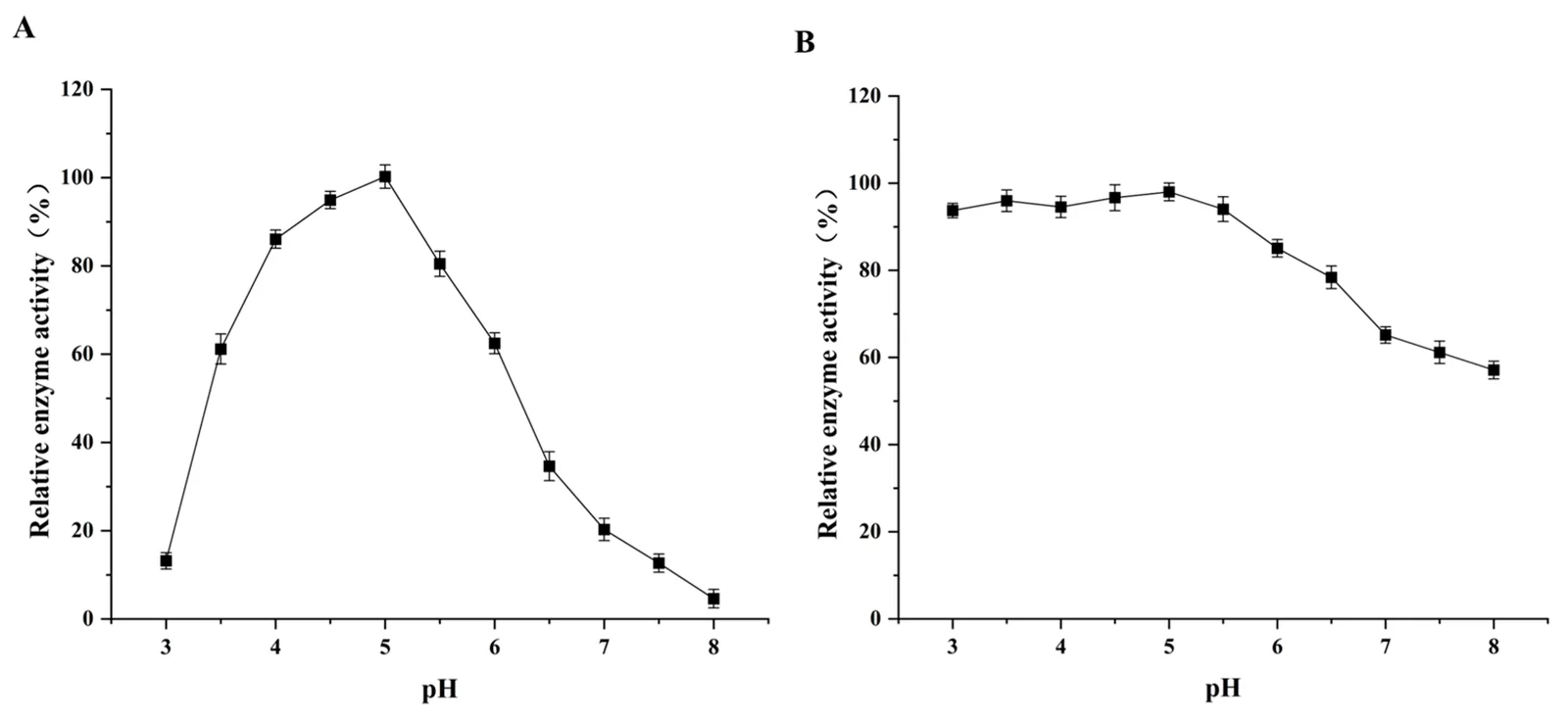
Effect of pH on DdpgA activity. (**A**) Optimal pH; (**B**) pH stability.

The experimental data were analyzed and response surface plots were constructed (Figure 5). Both the AB (induction temperature × IPTG concentration) and AC (induction temperature × induction time) interactions were significant (*p* < 0.01), as reflected by the steep temperature-dependent surfaces and the elliptical contour lines in Figure 6: the enzyme activity peaked at intermediate levels of the factors and declined markedly as the temperature rose toward 30 °C, indicating that neither adjusting the IPTG concentration nor prolonging the induction time could compensate for the adverse effect of an excessively high temperature. In contrast, the BC interaction was not significant (*p* = 0.3217), consistent with the comparatively flat IPTG concentration–induction time surface and its nearly circular contour lines, indicating that these two factors affected DdpgA production essentially independently.

The predicted optimal induction conditions for DdpgA expression in *E. coli* were an induction temperature of 24.5 °C, an IPTG concentration of 0.32 mmol/L, and an induction time of 20.5 h, under which the predicted intracellular enzyme activity was 330 U/mL. To verify the model, three replicate verification experiments were performed under the optimized conditions; the actual intracellular enzyme activity was 343.33 U/mL, with a relative deviation of 4.0% from the predicted value (<5%), indicating good agreement between the predicted and actual values and confirming the reliability of the response surface optimization. Compared with that before optimization (255.75 U/mL under the initial conditions), the intracellular enzyme activity of DdpgA increased by 34.3%, significantly improving the expression level of the recombinant enzyme.

### 3.4. Enzymatic Properties of DdpgA

#### 3.4.1. Optimal Reaction pH and pH Stability

The activity of the purified DdpgA was determined at 55 °C over a pH gradient of 3.0–8.0. The results showed that the optimal reaction pH of DdpgA was 5.0, classifying it as an acidic enzyme (Figure 6A). After treatment at pH 3.0–8.0 for 1 h, the residual activity of DdpgA remained above 80% at pH 3.0–6.0, indicating good stability of the enzyme under acidic conditions (Figure 6B).

#### 3.4.2. Optimal Reaction Temperature and Thermostability

**Figure 7:**
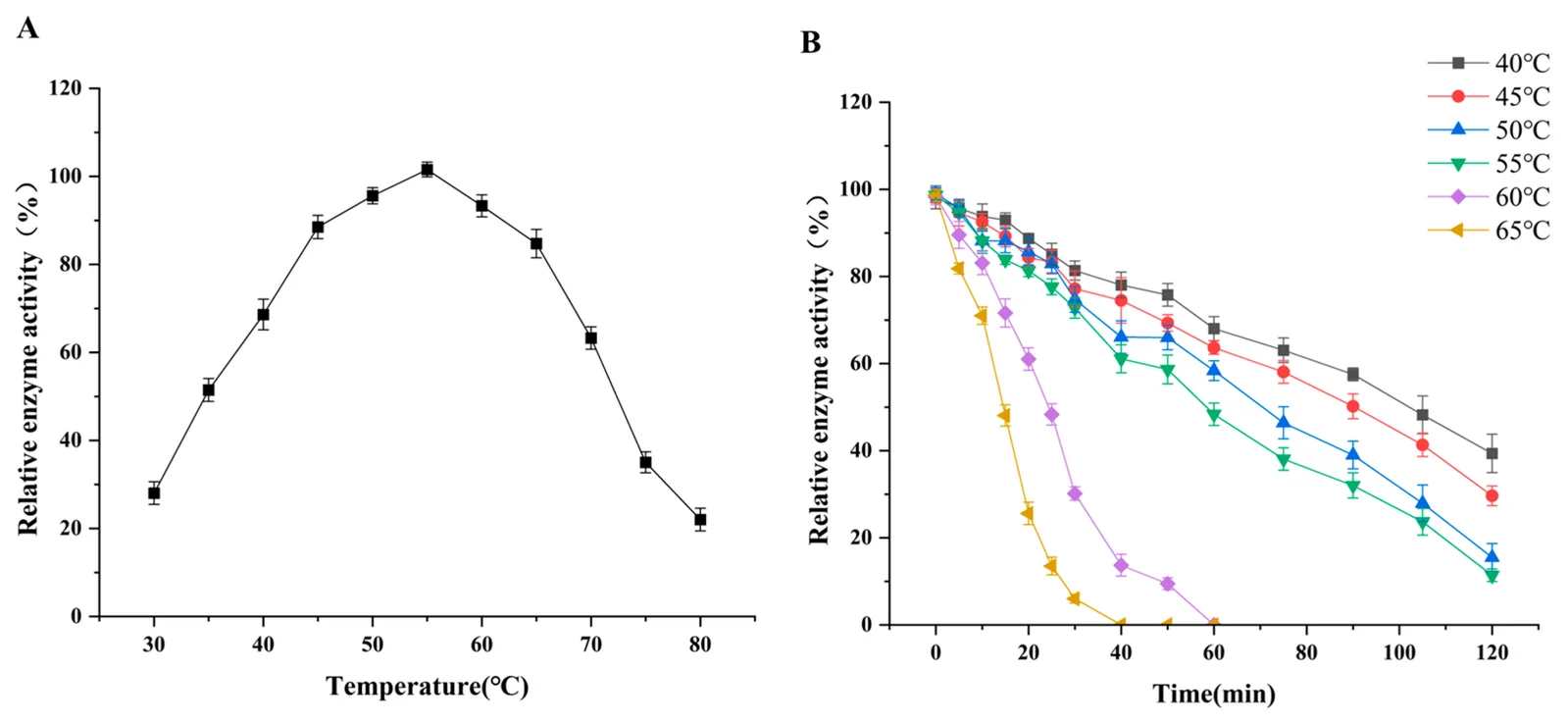
Effect of temperature on DdpgA activity. (**A**) optimal temperature; (**B**) stable temperature.

At the optimal pH, the enzyme activity was determined at different temperatures. The results showed that the optimal reaction temperature of DdpgA was 55 °C; within 30–55 °C, the activity increased significantly with temperature; the relative activity at 70 °C was approximately 63.3%, and it dropped rapidly to 35% at 75 °C (Figure 7A). DdpgA retained more than 50% of its activity after incubation at 55 °C for 60 min, showing good thermostability; after incubation at 60 °C and 65 °C for 30 min, the residual activities decreased to approximately 30.2% and 6%, respectively (Figure 7B). Therefore, DdpgA is an acidic mesophilic enzyme.

#### 3.4.3. Effects of Metal Ions and Chemical Reagents

**Figure 8:**
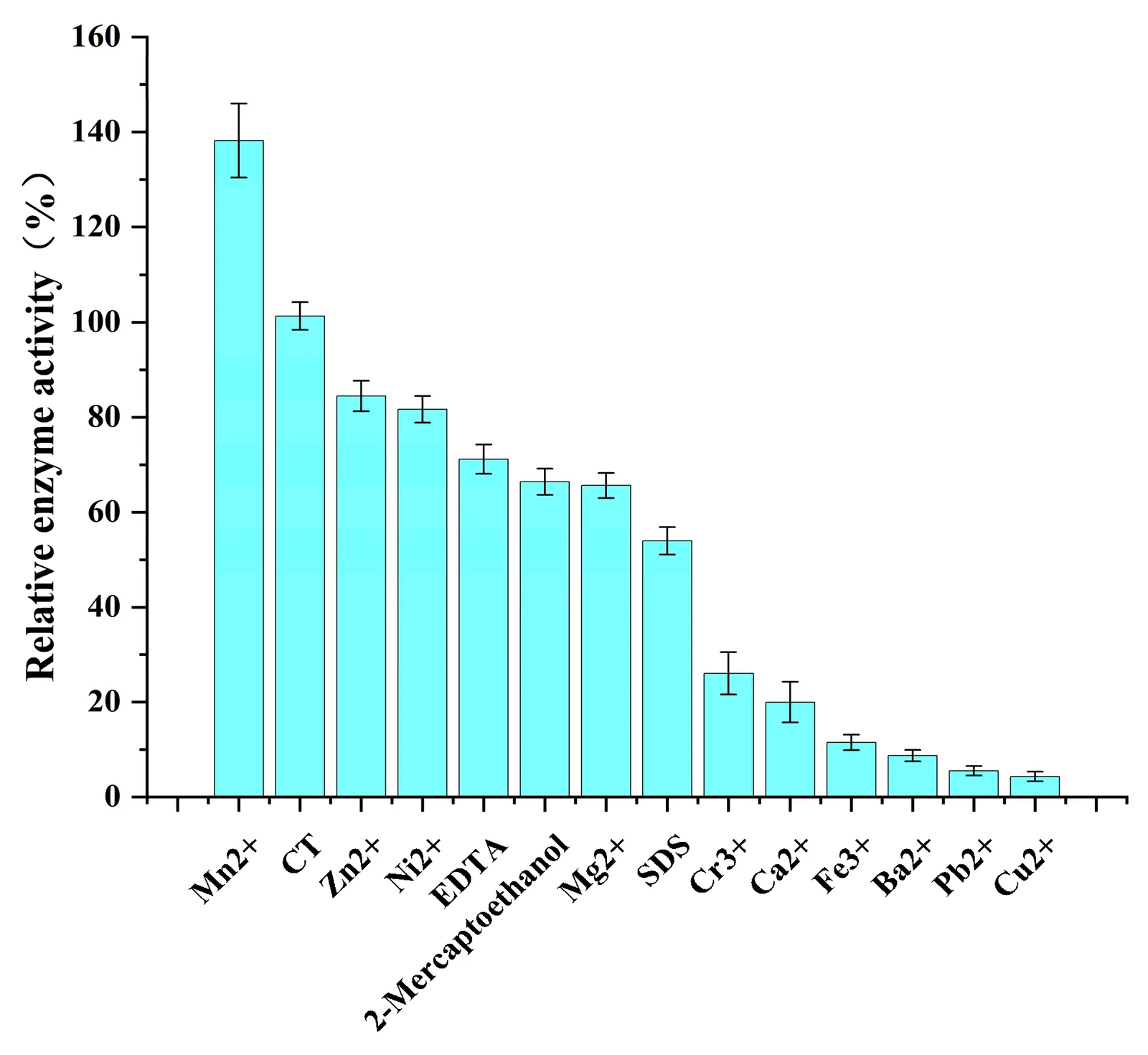
The effects of different metal ions and chemical reagents on the activity of DdpgA.

The effects of metal ions and chemical reagents on DdpgA activity were investigated (Figure 8). Mn^2+^ significantly activated the enzyme, with a relative activity of 138.2%. Ba^2+^, Pb^2+^, and Cu^2+^ were the strongest inhibitors, reducing the enzyme activity to below 10%. SDS almost completely inactivated the enzyme, EDTA reduced the activity to 71.15%, and β-mercaptoethanol treatment resulted in a relative activity of approximately 66.40%.

#### 3.4.4. Substrate Specificity

**Figure 9:**
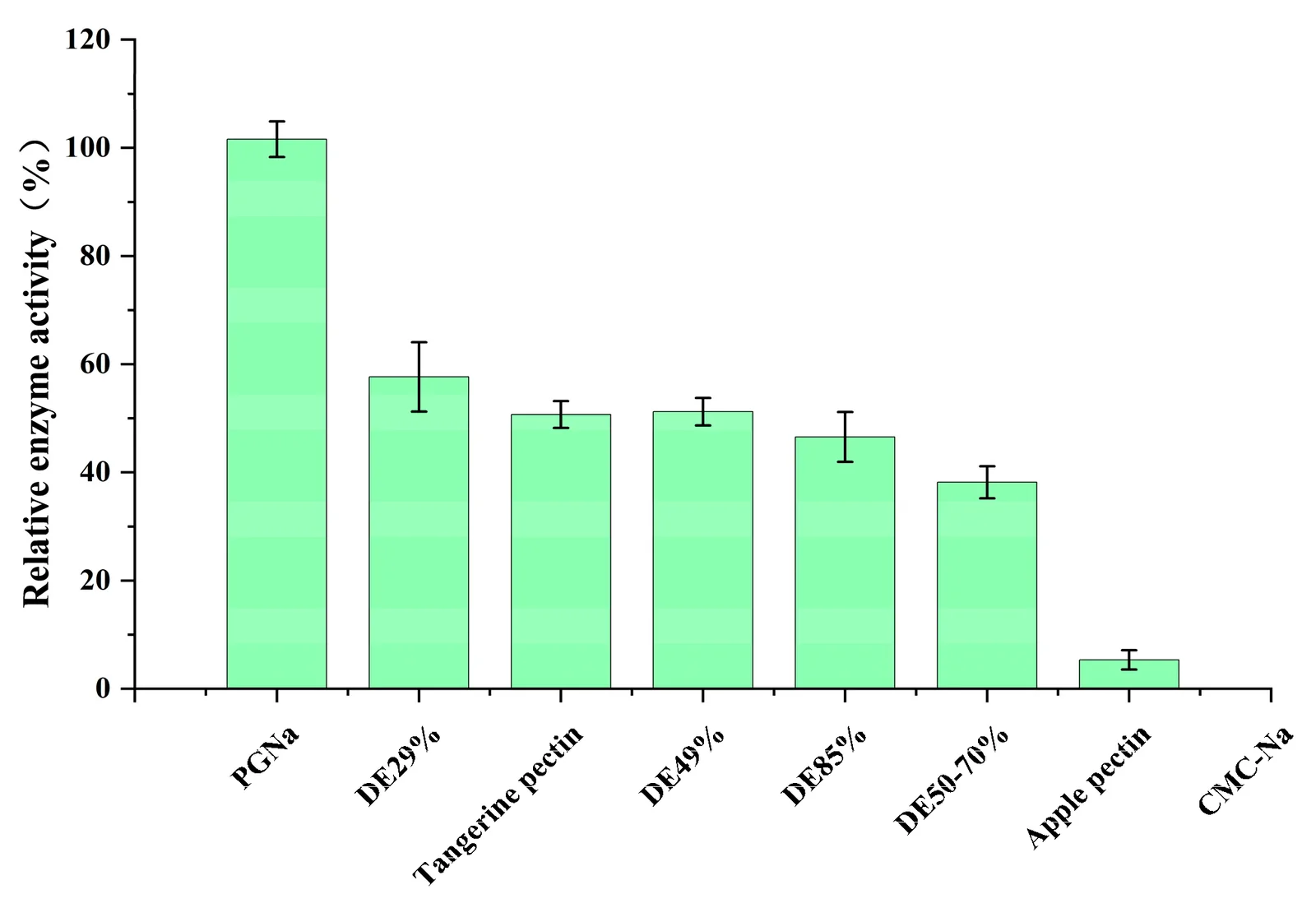
Effect of different substrates on DdpgA activity.

The substrate specificity of DdpgA was determined using sodium polygalacturonate as the reference (Figure 9). DdpgA exhibited the highest catalytic activity toward sodium polygalacturonate, consistent with its identity as a polygalacturonase. The relative activity toward low-esterification pectin (DE 29%) was approximately 57.65%, and the activity gradually decreased with increasing degree of esterification, dropping to approximately 46.55% for high-esterification pectin (DE ≥ 85%). Apple pectin was degraded the least (5.33%), and no activity was detected toward CMC-Na.

#### 3.4.5. Enzyme Kinetic Parameters

**Figure 10:**
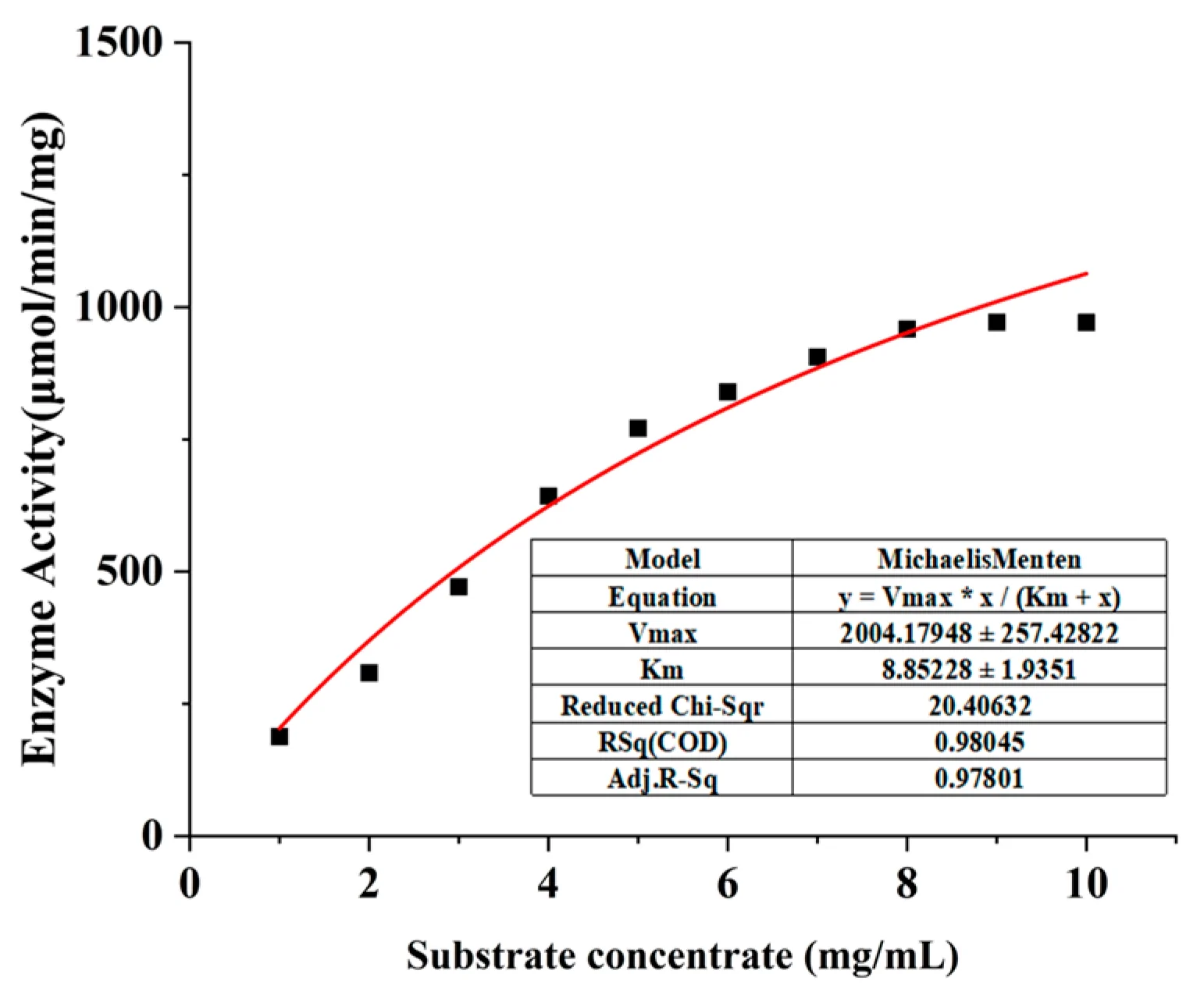
Michaelis–Menten kinetics of DdpgA to sodium polygalacturonate.

The apparent kinetic parameters of DdpgA were calculated by nonlinear fitting of the Michaelis–Menten equation (Figure 10, Table 4). The Km of DdpgA toward sodium polygalacturonate was 8.85 ± 1.94 mg/mL, the Vmax was 2004.18 ± 257.43 μmol/min/mg, the kcat was 2146 ± 276 s^−1^, and the catalytic efficiency (kcat/Km) was 242 ± 61 mL/(mg·s).

#### 3.4.6. MALDI-TOF MS Analysis of the Hydrolysis Products

**Figure 11:**
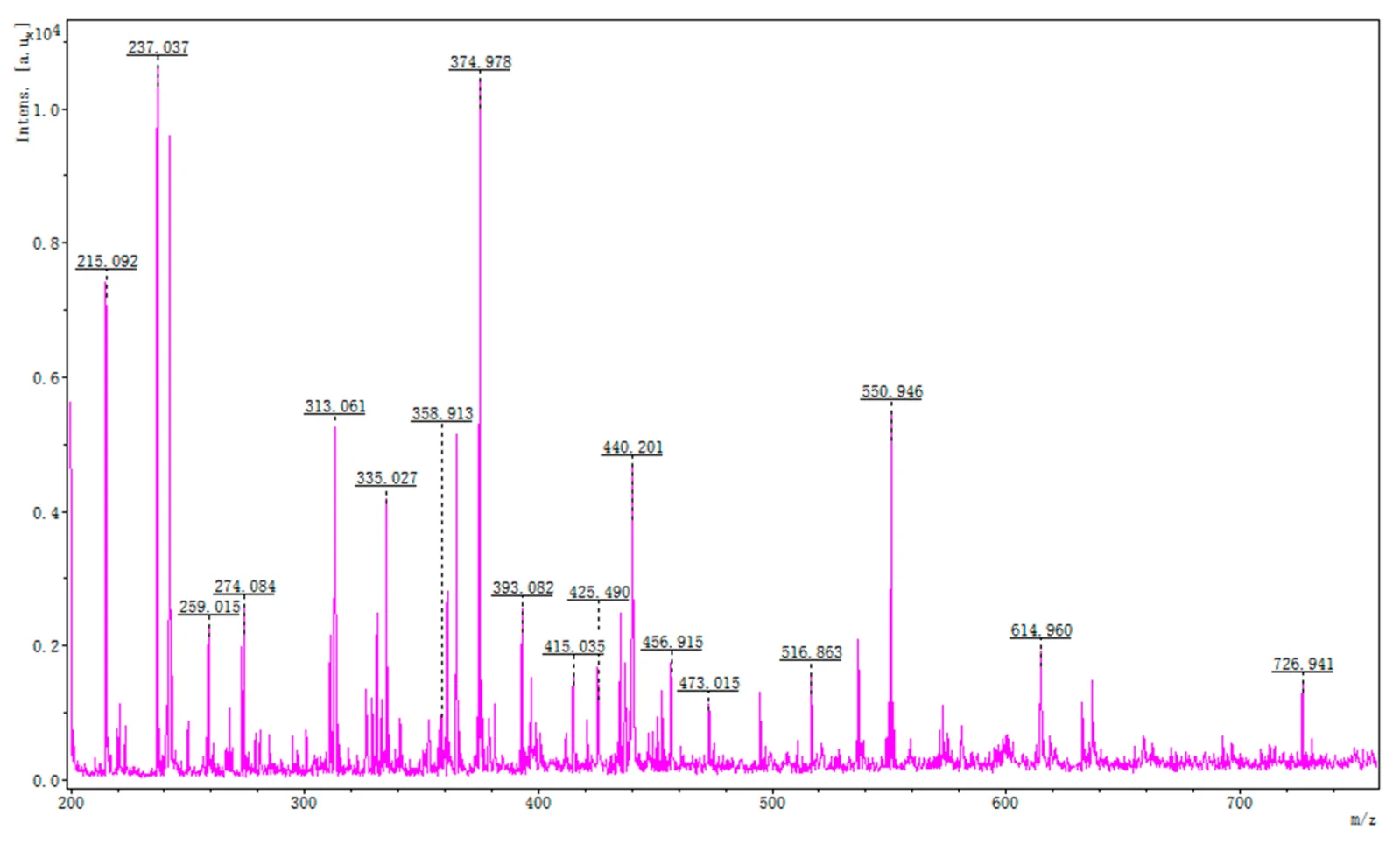
MALDI-TOF MS analysis of DdpgA enzymatic hydrolysis products.

The hydrolysis products of DdpgA were analyzed by MALDI-TOF MS (Figure 11). A prominent product peak appeared at *m*/*z* 374.98, corresponding to the dehydrated sodium adduct of a galacturonic acid dimer, [GalA_2_−H_2_O+Na]^+^ (theoretical value: 375.05 Da). The predominance of dimeric products is characteristic of exo-polygalacturonases that release digalacturonic acid from the substrate termini, indicating that DdpgA is an exo-digalacturonase-type polygalacturonase by the hydrolysis products, consistent with above homology analysis predictions.

### 3.5. Application of DdpgA in the Bioscouring of Ramie Fabric

#### 3.5.1. Enzyme Dosage on Scouring Effect

**Figure 12:**
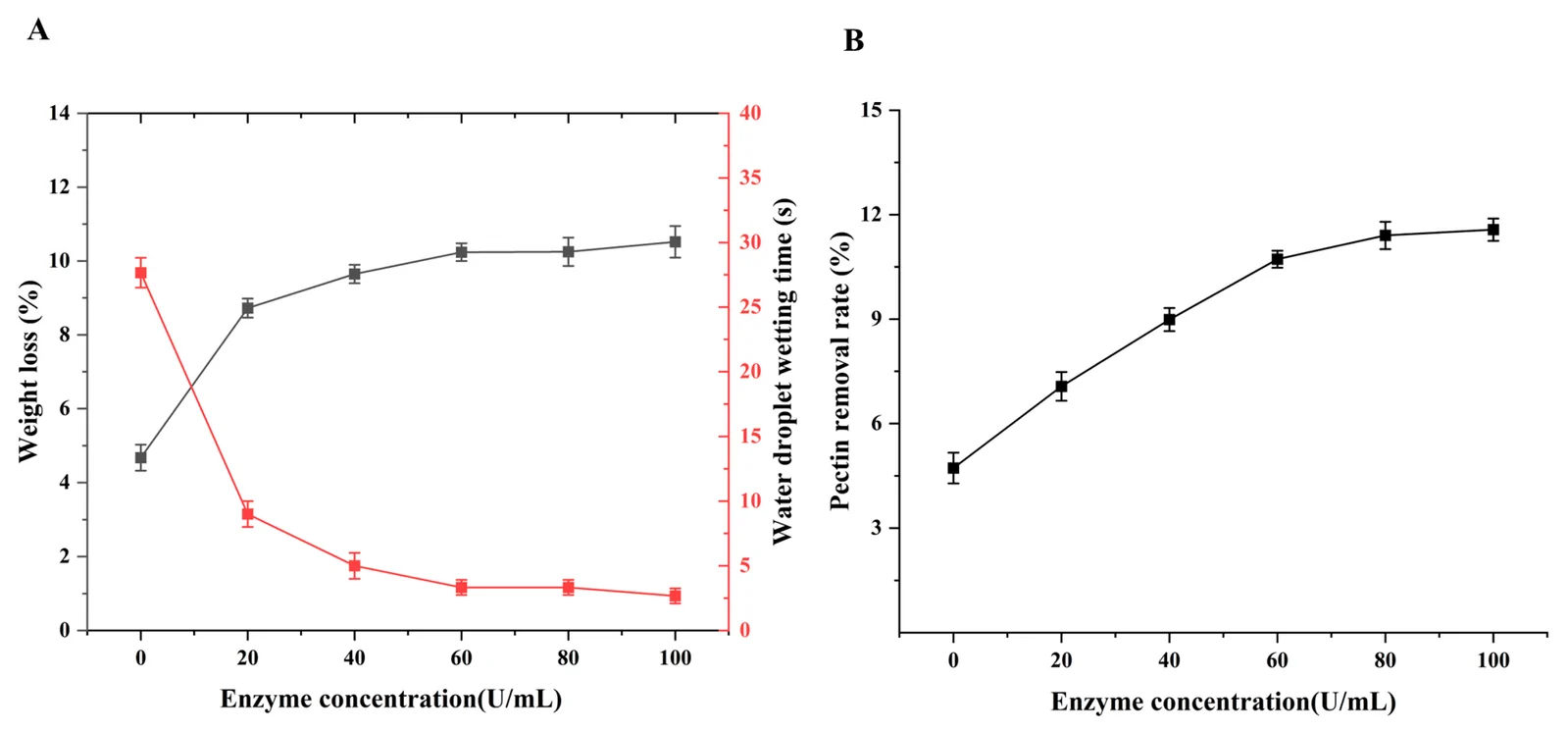
Effects of enzyme dosage on the scouring of ramie fabric. (**A**) Weight loss rate and water-drop wetting time; (**B**) pectin removal rate.

The effect of the dosage of DdpgA alone on the scouring of ramie fabric was investigated at pH 6.0 and 50 °C for 1 h with a bath ratio of 1:15 (Figure 12). The pectin removal rate and wettability increased rapidly with increasing enzyme concentration and then plateaued. At an enzyme dosage of 60 U/mL, the weight loss rate was 10.23% and the pectin removal rate was 10.72%, while the water-drop wetting time was shortened from 27 s to less than 4 s. Increasing the dosage to 100 U/mL produced markedly smaller improvements, indicating that the enzymatic reaction was approaching saturation.

#### 3.5.2. Treatment Time on Scouring Effect

**Figure 13:**
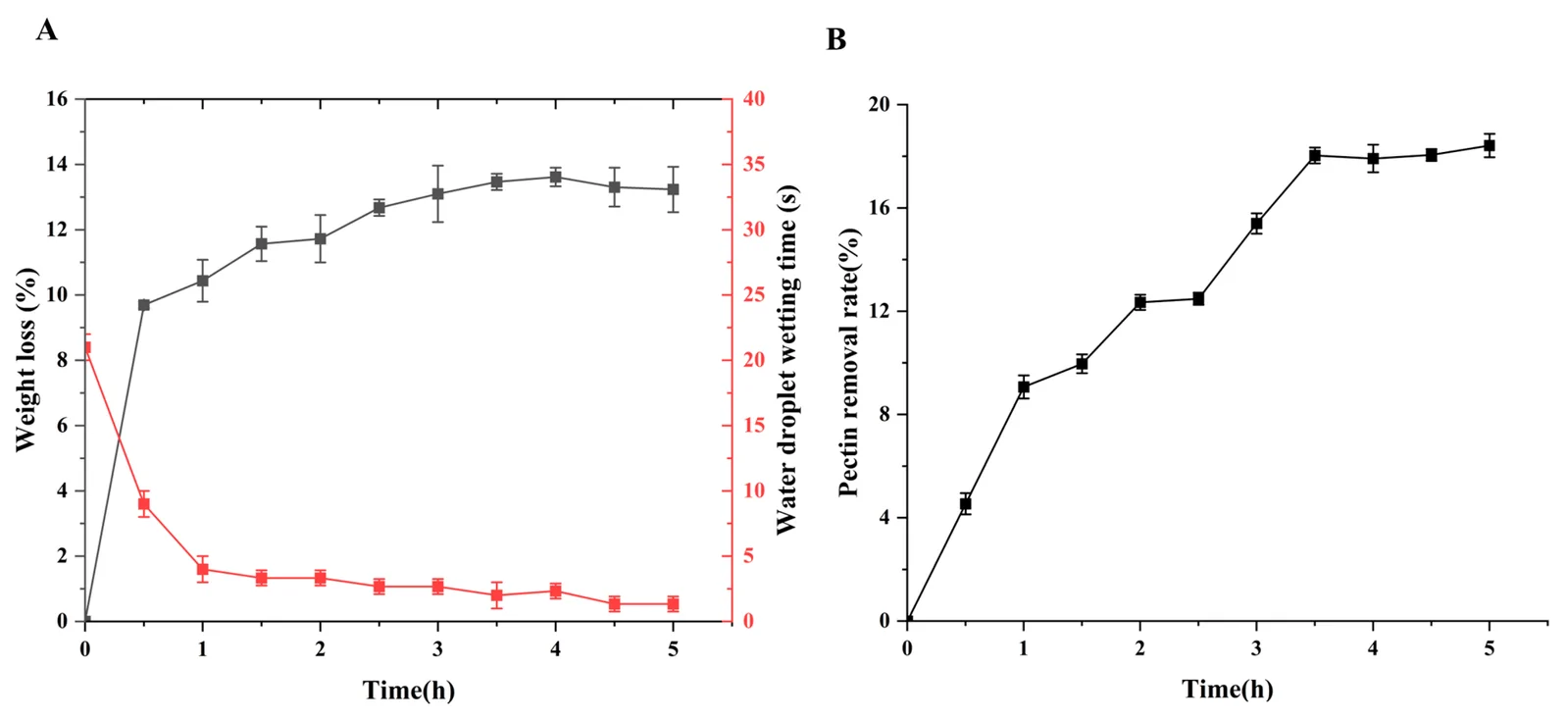
Effects of treatment time on the scouring of ramie fabric. (**A**) Weight loss rate and water-drop wetting time; (**B**) pectin removal rate.

The effect of the DdpgA treatment time (0.5–5 h) on the scouring of greige ramie fabric was investigated at an enzyme dosage of 60 U/mL, pH 6.0, and 50 °C with a bath ratio of 1:15 (Figure 13). With prolonged treatment, the pectin removal rate and weight loss rate increased and the wetting time decreased. After 1 h, the weight loss rate was 10.43%, the pectin removal rate was 9.07%, and the wetting time was less than 4 s; after 3.5 h, the weight loss rate was 13.46%, the pectin removal rate was 18.03%, and the wetting time was less than 3 s, indicating that the enzymatic reaction had essentially reached equilibrium; beyond 5 h, the improvement slowed. Considering the treatment efficiency and economic cost, 3.5 h was selected as the appropriate treatment time.

#### 3.5.3. Comparison of Compound-Enzyme Scouring and Conventional Alkaline Scouring

**Figure 14:**
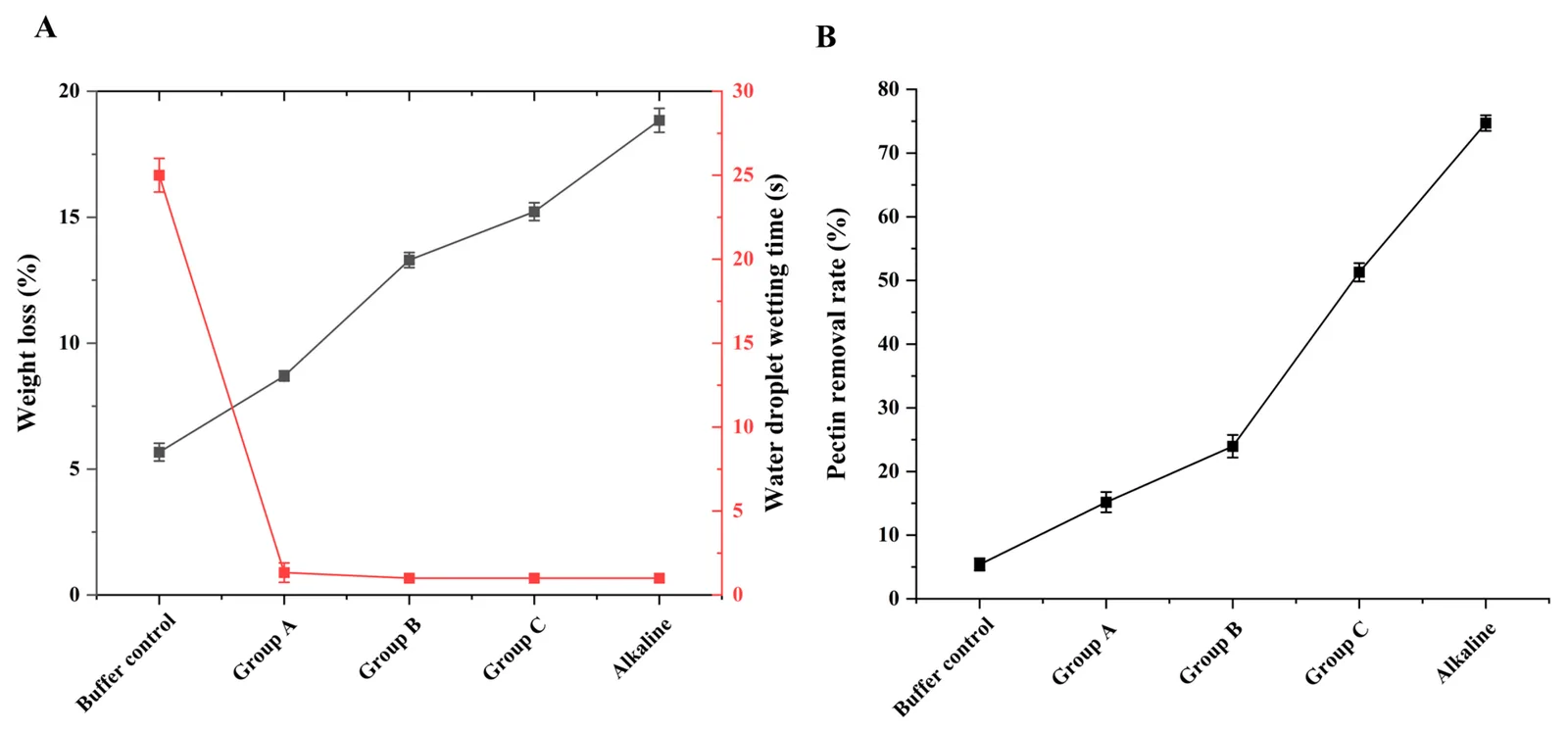
Comparison of composite enzymatic scouring and conventional alkaline scouring of ramie fabric. (**A**) Weight loss rate and water-drop wetting time; (**B**) pectin removal rate. Buffer control, buffer (pH 6.0) only; Group A, mannanase and xylanase (1:1); Group B, mannanase, xylanase and commercial polygalacturonase (1:1:1); Group C, mannanase, xylanase and DdpgA (1:1:1); Alkaline, conventional alkaline scouring (NaOH 8 g/L, 95 °C, 90 min). All enzymatic treatments were carried out at an enzyme loading of 60 U/mL, pH 6.0 and 50 °C for 3.5 h.

The weight loss rates and pectin removal rates of the ramie fabrics after the different treatments were as follows: commercial polygalacturonase compound enzyme (13.30% vs. 23.95%), DdpgA compound enzyme (15.22% vs. 51.28%), and conventional alkaline scouring (18.84% vs. 74.70%); the wetting times of all enzymatic treatment groups were less than 2 s (Figure 14). The pectin removal rate achieved with DdpgA was significantly higher than that achieved with the commercial polygalacturonase but remained lower than that of alkaline scouring.

#### 3.5.4. Apparent Characteristics of Ramie Fabric

**Figure 15:**
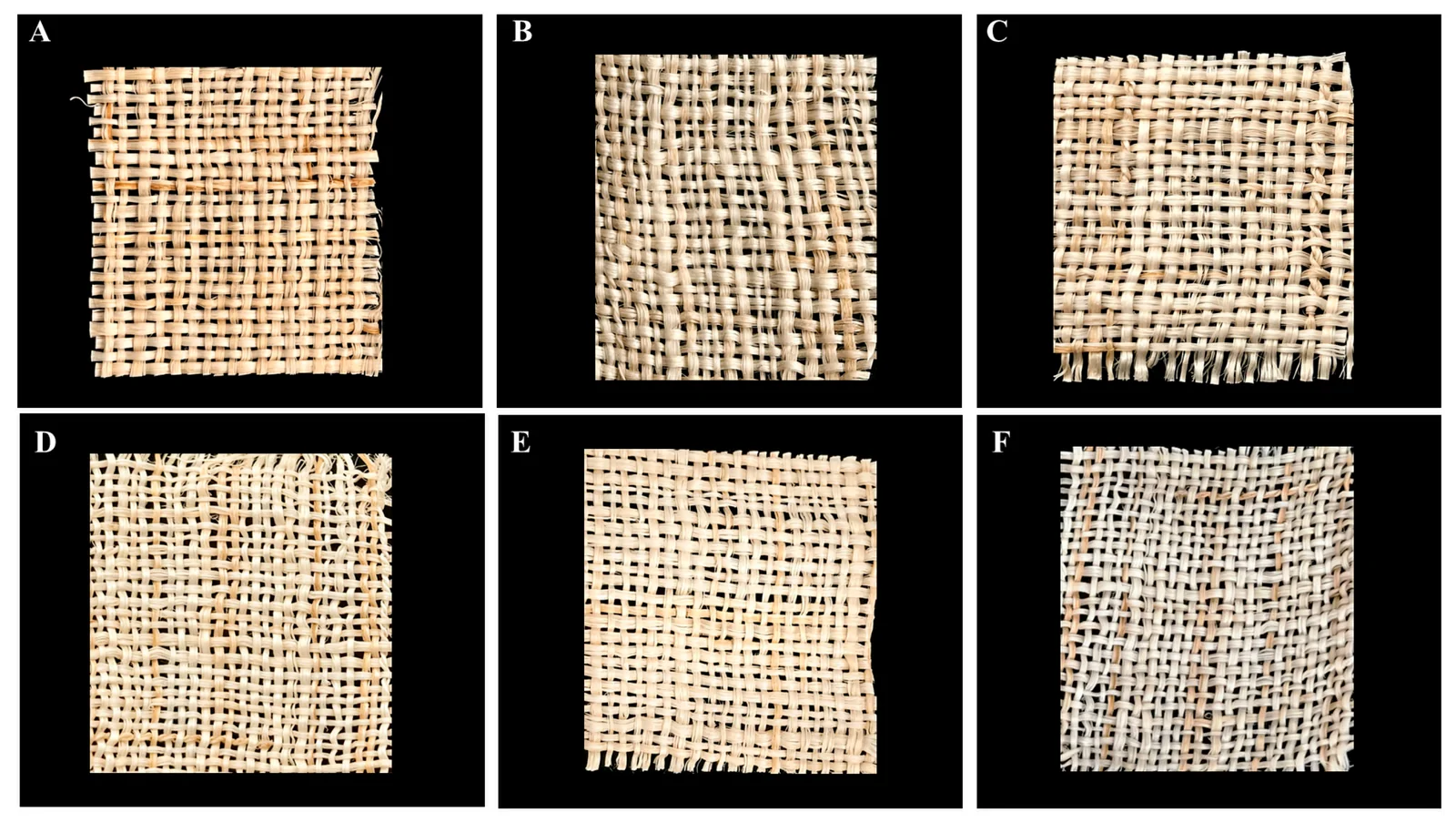
Apparent characteristics of ramie fabric. (**A**) Untreated control; (**B**) Buffer control; (**C**) Group A; (**D**) Group B; (**E**) Group C; (**F**) Alkaline. Untreated control:raw ramie fabric; other treatments are identical to those described in Section 3.5.3.

The apparent characteristics of the greige ramie fabrics after the different scouring treatments were recorded (Figure 15). The untreated ramie fabric was dull yellow-brown with blurred warp and weft textures, and no obvious change was observed in the buffer control. The color of the two-enzyme group was slightly lightened, the commercial polygalacturonase group was partially off-white, and the DdpgA group was uniformly light off-white with a clean fabric surface and clear textures; the whiteness and cleanliness of the DdpgA group were superior to those of the former two groups, consistent with the weight loss and pectin removal results (Group C > Group B > Group A). The alkaline-scoured group was the whitest, and its apparent improvement was better than that of all the enzymatic treatment groups.

**Figure 16:**
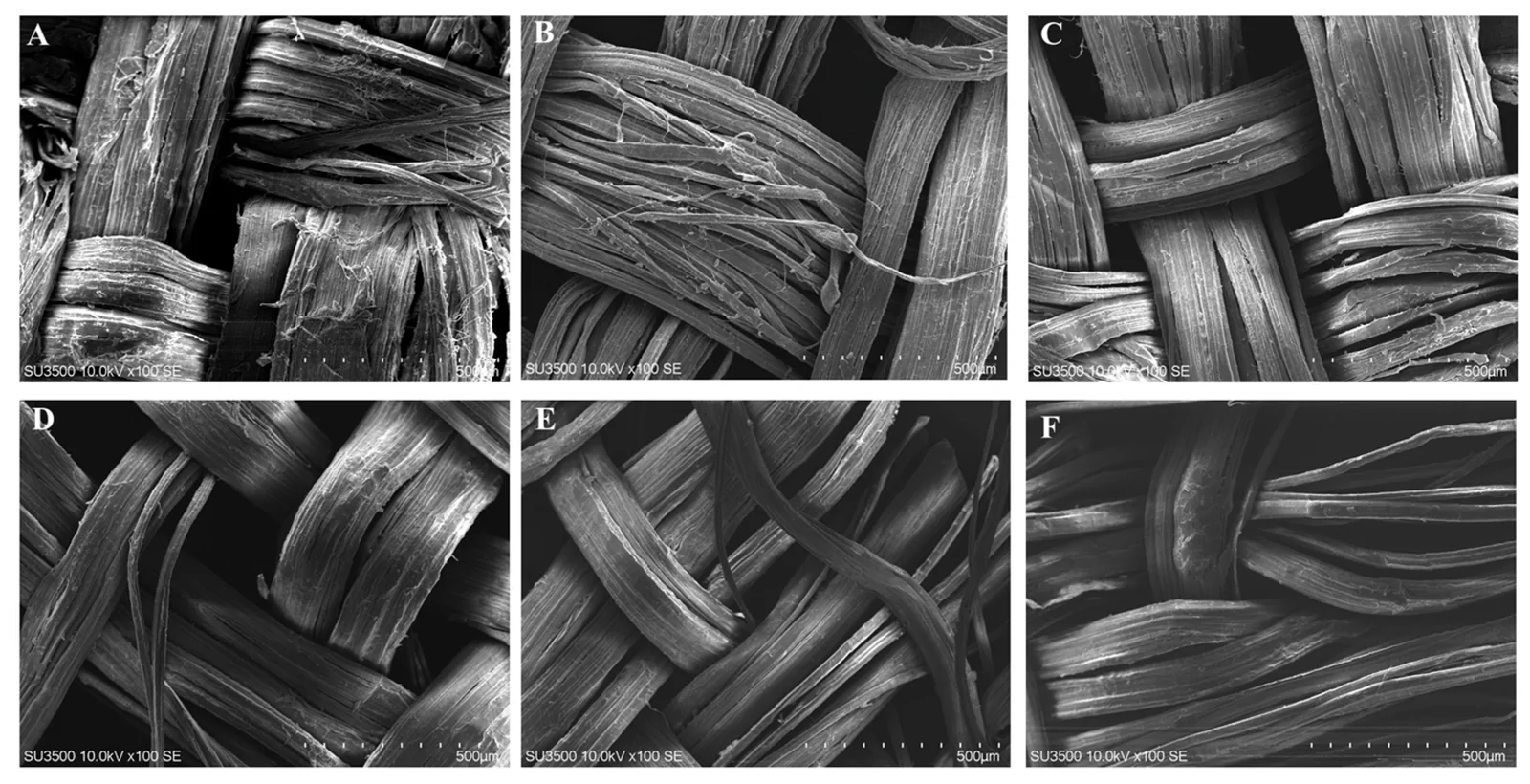
SEM micrographs of ramie fabric surfaces before and after scouring. (**A**) Untreated control; (**B**) Buffer control; (**C**) Group A; (**D**) Group B; (**E**) Group C; (**F**) Alkaline.

The morphological changes on the fiber surface were observed by SEM (Figure 16). The fibers of the untreated ramie fabric and the buffer control were rough, with abundant gummy and waxy substances and adhered fiber bundles. In the two-enzyme group, the gum was only partially loosened. In the commercial polygalacturonase group, some single fibers were exposed but remained adherent. In the DdpgA group, the gummy and waxy substances were effectively removed, the surface was smooth, the outlines of the single fibers were clear, and the degree of bundle separation was improved, outperforming the former two groups. The alkaline-scoured group was the cleanest with the highest degree of dispersion; however, the strong alkali and high temperature involved entail high energy consumption, substantial alkaline wastewater discharge, and easy fiber damage. In contrast, compound-enzyme scouring under mild conditions removes gummy matter effectively, as reflected by the shortened wetting time and cleaner fiber surface, while avoiding the fiber damage associated with strong alkali and high temperature.

## 4. Discussion

**Figure 17:**
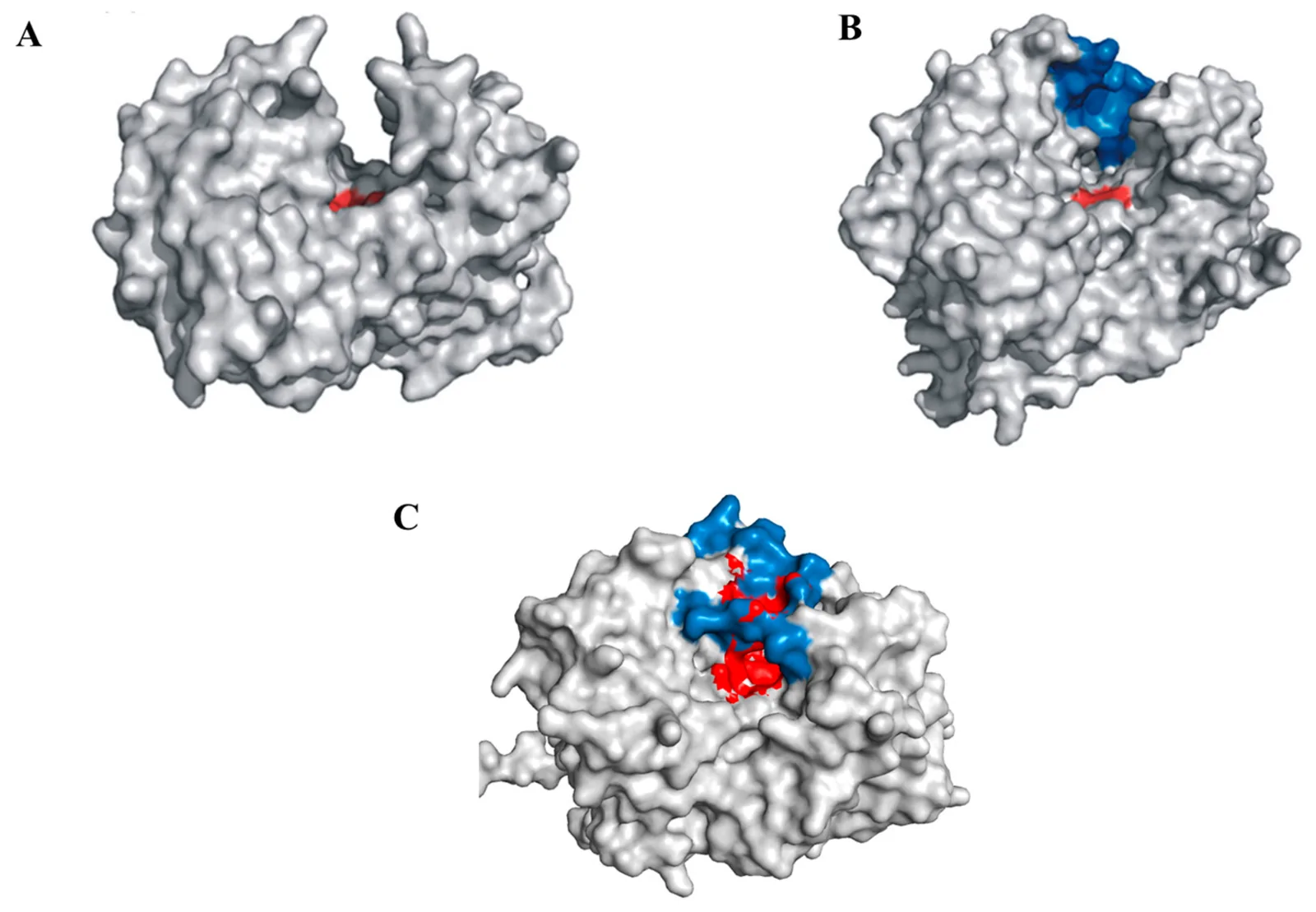
Comparison of endo-galactosidase and exo-galactosidase. (**A**) Endo-galactosidase EcGH28. (**B**) Exo-galactosidase YeGH28. (**C**) DdpgA. The catalytic center is marked in red shading; the loop structure is marked in blue shading.

In this study, homology modeling and molecular docking showed that the galacturonic acid dimer was stably embedded in the catalytic pocket of DdpgA, with a binding energy of approximately −8.8 kcal/mol, which is stronger than the typical docking levels reported for GH28 enzymes (−6 to −8 kcal/mol) and consistent with its experimentally observed high substrate affinity [23,24]. The main hydrolysis product of DdpgA was digalacturonic acid, and DdpgA was most closely related to YeGH28, an exo-digalacturonase-type polygalacturonase from *Yersinia enterocolitica* (PDB: 2UVE; PDB: 2UVF) [25,26]; DdpgA may therefore be an exo-digalacturonase-type polygalacturonase. Both DdpgA and YeGH28 contain an FN3 domain at the N-terminus, which is not directly involved in catalysis but may stabilize the adjacent active-site loops to assist substrate binding and maintain the overall stability of the enzyme [25,26]. Analysis of the catalytic pockets of polygalacturonases shows that, in exo-polygalacturonases (Figure 17), a loop structure blocks the reducing end of the active center. Endo-polygalacturonases also possess loops in the active pocket, but their conformation opens toward the periphery of the active center, forming the wall of the catalytic channel. The additional loop of exo-polygalacturonases forms a unique “closed” structure, and this topological arrangement hinders the complete binding of the substrate to the enzyme, constituting an exo-acting mode [25,26]. In addition, the catalytic mechanisms of glycoside hydrolases mainly include retaining and inverting mechanisms [27]. In the retaining mechanism, the two acidic amino acids catalyze the reaction through double-displacement involving a covalent glycosyl-enzyme intermediate, and the carboxyl groups of these two acidic residues are approximately 5.5 Å apart. In the inverting mechanism, the carboxyl groups are approximately 9.5 Å apart, and catalysis proceeds through a single-displacement reaction. The distances among the catalytic triad Asp374-Asp395-Asp396 of DdpgA are all within 5.5 Å, suggesting that DdpgA does not adopt the canonical inverting mechanism.

The outstanding advantages of DdpgA lie in its high specific activity, high expression level, and excellent catalytic efficiency (Table 5). Its specific activity reached 523.3 U/mg and the shake-flask crude-enzyme activity 343.33 U/mL, substantially higher than those of most exo-PGs—such as the rumen IDSPGA28-4/16 (31.2 and 330.4 U/mg) [24] and AfumExoPG28A (5.72 U/mg) [28]—although below the most active endo-PGs, such as the A. aculeatus EndoPG (1892.1 U/mg on citrus pectin) [29]; consistent with its exo-digalacturonase character and FN3-bearing architecture, it aligns most closely with YeGH28 [25,26] rather than the processive rumen enzymes that release GalA monomers [24]. Its Vmax toward sodium polygalacturonate was 2004.18 ± 257.43 μmol/min/mg (vs. 23.6 U/mg for AfumExoPG28A), and its Km of 8.85 ± 1.94 mg/mL was markedly lower than 25.4 mg/mL, combining high substrate affinity with a very high turnover rate. DdpgA is an acidic, mesophilic-to-thermotolerant enzyme (opt. pH 5.0 and 55 °C; stable at pH 3.0–6.0 and 55 °C), whose temperature optimum lies above those of the rumen exo-PGs (40 °C) and EndoPG (50 °C), matches the recently reported TL-PG1 (55 °C) [30], and remains below the more thermophilic AfPGA (70 °C) [31]. Although AfPGA tolerates higher temperatures, the catalytic activity of DdpgA is far higher (523.3 vs. 52.6 μmol/min/mg); DdpgA is therefore an enzyme with considerable application potential under medium- and low-temperature conditions, particularly for low-temperature textile scouring. Metal ions exerted a strong, enzyme-specific influence: Mn^2+^ significantly activated DdpgA, whereas Ba^2+^, Pb^2+^, and Cu^2+^ strongly inhibited it, the reverse of EndoPG, in which Cu^2+^ stimulates and Mn^2+^ inhibits [29]; manganese may accordingly be supplemented to boost performance, while the heavy-metal content of the process water must be strictly controlled for each enzyme preparation.

The gummy substances on the surface of ramie fabric are composed mainly of low-esterification pectin, accompanied by hemicelluloses such as mannan and xylan coating the fiber surface [32]. DdpgA prefers low-esterification pectin and maintains high activity under weakly acidic conditions (pH 6.0), and it showed no degrading activity toward CMC-Na; i.e., it does not damage cellulose, meeting the core safety requirement for PGs used in textile scouring and conforming to the selection principle of an efficient scouring enzyme system featuring ‘high polygalacturonase activity and extremely low cellulase activity’ [33,34,35]. In compound-enzyme scouring, the group supplemented with DdpgA exhibited significantly higher pectin removal and weight loss rates than the group with an equal amount of commercial polygalacturonase, with synchronous improvements in apparent whiteness and SEM morphology, confirming the central role of polygalacturonase in the scouring enzyme system: microbial community studies of bast-fibre retting have shown that polygalacturonase activity and the population of pectin-degrading bacteria rise in close parallel with pectin removal and are strongly correlated with the degumming rate [36]; endo-PG treatment can yield fully spinnable fibers [37]; and fiber strength obtained by hemicellulase–pectinase synergy has been reported to be comparable to or better than that from alkali treatment [38]. Compared with conventional alkaline scouring, the enzymatic approach still lags in pectin removal rate; however, alkaline scouring requires strong alkali at 95 °C, with high energy consumption, and a tendency to damage fibers [32,38]. In contrast, the enzymatic method operates under mild conditions, does not damage the cellulose backbone, and generates less wastewater pollution, which is aligned with the direction of green manufacturing in the textile industry; recent studies on the synergy between polygalacturonase and oxidation systems have also shown that mild processes can gradually approach the effect of alkaline scouring [39,40,41]. The gap with alkaline scouring could be further narrowed by adding penetrating agents, optimizing the enzyme formulation, enzyme molecular modification, and adjusting the enzyme dosage and treatment time [42].

## 5. Conclusions

The polygalacturonase gene *ddpg*A was mined from the genome of the highly effi-cient bast-fiber-degumming strain *D. dadantii* DCE-01 and heterologously expressed. The recombinant enzyme combines high catalytic activity, a broad acidic pH tolerance, mesophilic stability, and cellulose-safe action, and its activity is selectively modulated by metal ions: Mn^2+^ enhanced it to 138.2%, whereas Ba^2+^, Pb^2+^, and Cu^2+^ reduced it to below 10%, suggesting appropriate Mn^2+^ supplementation and strict control of heavy metals in the process water. When applied to ramie fabric, the compound-enzyme process achieved alkali-comparable wetting under far milder conditions, retaining fiber strength with lower energy consumption and alkaline wastewater discharge, at the cost of lower pectin removal, weight loss, and whiteness and a longer treatment time. Accordingly, DdpgA-based bioscouring is best regarded at this stage as an eco-friendly, strength-preserving complement to, rather than a one-step replacement for, alkaline scouring. This work provides a novel gene resource for pectinase preparations dedicated to bast-fiber bioscouring and a basis for understanding the structural determinants of activity differences among GH28 polygalacturonases; narrowing the remaining performance gap through formulation optimization, penetrating agents, and oxidative–enzymatic synergy will be the focus of future work.

## Figures and Tables

**Table 1:** Predicted physicochemical properties of DdpgA.

Gene ID	Amino Acids (AA)	Signal Peptide (AA)	MW (kDa)	N-Glycosylation	O-Glycosylation	Disulfide Bonds	pI	Aliphatic Index
DdpgA	602	27	64.25	6	11	1	7.14	82.99

**Table 2:** Response surface design and results.

Run	Induction Temperature (°C)	IPTG Concentration (mmol/L)	Induction Time (h)	Intracellular Enzyme Activity (U/mL)
1	20	0.5	24	258.78
2	30	0.5	24	255.83
3	25	0.7	24	275.99
4	30	0.7	20	260.16
5	25	0.3	16	315.02
6	25	0.7	16	285.74
7	30	0.5	16	263.32
8	25	0.5	20	319.41
9	25	0.5	20	320.74
10	25	0.5	20	330.25
11	20	0.7	20	210.18
12	25	0.5	20	320.97
13	20	0.5	16	210.25
14	20	0.3	20	270.77
15	30	0.3	20	265.28
16	25	0.5	20	316.54
17	25	0.3	24	320.82

**Table 3:** Analysis of variance (ANOVA) for the regression model.

Source	Sum of Squares	df	Mean Square	F-Value	*p*-Value	Significance
Model	22,973.96	9	2552.77	48.02	<0.0001	**
A-Induction temperature	1118.88	1	1118.88	21.05	0.0025	**
B-IPTG concentration	2443.70	1	2443.70	45.96	0.0003	**
C-Induction time	171.96	1	171.96	3.23	0.1151	
AB	769.23	1	769.23	14.47	0.0067	
AC	784.56	1	784.56	14.76	0.0064	
BC	60.45	1	60.45	1.14	0.3217	
A^2^	15,752.76	1	15,752.76	296.30	<0.0001	**
B^2^	327.44	1	327.44	6.16	0.0421	*
C^2^	752.77	1	752.77	14.16	0.0070	**
Residual	372.15	7	53.16			
Lack of Fit	265.79	3	88.60	3.33	0.1377	
Pure Error	106.36	4	26.59			
Cor Total	23,347.11	16				

** *p* < 0.01, * *p* < 0.05.

**Table 4:** Kinetic parameters of DdpgA.

Kinetic Parameters	DdpgA
Km (mg/mL)	8.85 ± 1.94
Vmax (μmol/min/mg)	2004.18 ± 257.43
kcat (s^−1^)	2146 ± 276
kcat/Km(mL/(mg·s))	242 ± 61

**Table 5:** Alignment of DdpgA with representative recombinant GH28 polygalacturonases.

Enzyme	Source	Type	Opt. pH	Opt. T (°C)	Enzyme Activity (U/mg)
DdpgA	*Dickeya dadantii DCE-01*	Exo-PG	5.0	55	523.3
IDSPGA28-4	*Sheep rumen bacterium*	Exo-PG	5.0	40	31.2
IDSPGA28-16	*Sheep rumen bacterium*	Exo-PG	5.0	40	330.4
EndoPG	*Aspergillus aculeatus*	Endo-PG	5.0	50	1892.1
TL-PG1	*Thermomyces lanuginosus*	n.r.	6.0	55	n.r. (462.6 U/mL)
AfPGA	*Aspergillus fumigatus HBFH5*	n.r.	5.0	70	52.6

n.r., not reported.

## Data Availability

The original contributions presented in this study are included in the article. Further inquiries can be directed to the corresponding authors.
